# Neuroprotective potential of a TrkB-FL-derived cell-penetrating peptide in cochlear synaptopathy and noise-induced hearing loss

**DOI:** 10.1186/s10020-026-01539-9

**Published:** 2026-06-29

**Authors:** Elena Torres-Campos, Isabel Varela-Nieto, Margarita Díaz-Guerra

**Affiliations:** 1 Instituto de Investigaciones Biomédicas Sols-Morreale (IIBM), Consejo Superior de Investigaciones Científicas-Universidad Autónoma de Madrid (CSIC-UAM), Arturo Duperier, 4, Madrid, 28029 Spain; 2 Centro de Investigación Biomédica en Red en Enfermedades Raras (CIBERER), Instituto de Salud Carlos III (ISCIII), Madrid, 28029 Spain; 3 Instituto de Investigación Sanitaria del Hospital La Paz (IdiPAZ), Madrid, 28046 Spain

**Keywords:** Cell-penetrating peptides, Cochlear synaptopathy, Excitotoxicity, Neuroprotection, Neurotrophic system, Hearing loss

## Abstract

**Background:**

Noise-induced hearing loss (NIHL) is the second leading cause of deafness globally. However, effective pharmacological treatments remain unavailable. Excitotoxicity, an early NIHL event, is a central mechanism in degeneration of cochlear synapses established between inner hair cells (IHCs) and spiral ganglion neurons (SGNs). This excitotoxic damage can trigger the degradation of the neurotrophic TrkB receptor, thereby inhibiting brain-derived neurotrophic factor (BDNF) signaling and challenging neurotrophin-based therapies for hearing loss.

**Methods:**

We employed an ex vivo model of excitotoxicity, where excitotoxicity was induced in cochlear explants by overstimulation of excitatory glutamate receptors, and an in vivo model of noise overexposure. The effects of excitotoxicity on the neurotrophic system and cell architecture were established by immunohistochemistry. Hearing function was evaluated in vivo by the auditory brainstem responses (ABR) test, performed before and after noise overexposure.

**Results:**

In this study, we evaluated the therapeutic potential in the inner ear of MTFL_457_, a cell-penetrating peptide designed to prevent TrkB-FL degradation in brain excitotoxicity. MTFL_457_ showed efficient distribution across cochlear cell types in both ex vivo and in vivo models, supporting its ability to reach the inner ear and suggesting that it can cross the blood-labyrinth barrier after systemic administration. In explants undergoing excitotoxicity, MTFL_457_ prevented TrkB-FL dysregulation, partially restored downstream prosurvival signaling, and significantly reduced neuronal damage and features associated with cochlear synaptopathy. In vivo, despite known sex-dependent differences in susceptibility to noise-induced damage, treatment with MTFL_457_ preserved auditory function and synaptic integrity in both males and females, although the magnitude of protection varied between sexes. Together, these findings support a protective effect of MTFL_457_ in models of excitotoxic cochlear injury.

**Conclusions:**

These results support the therapeutic potential of peptide MTFL_457_ for treatment of NIHL and possibly other types of sensorineural hearing loss likewise associated with excitotoxicity.

**Supplementary Information:**

The online version contains supplementary material available at https://doi.org/10.1186/s10020-026-01539-9.

## Introduction

Noise exposure, whether acute or prolonged, poses a significant threat to auditory health (Lie et al. 2016). Indeed, noise-induced hearing loss (NIHL) is the second most common cause of deafness after presbycusis, affecting around 5% of the global population, although this figure is likely underestimated (Chadha et al. 2021; Natarajan et al. 2023). Alarmingly, over one billion young adults are at risk of developing NIHL due to unsafe recreational use of audio devices (Szibor et al. 2018). Currently, there are no pharmacological treatments to prevent or repair local noise-induced damage, underscoring the urgent need to develop innovative therapeutic approaches (Varela-Nieto et al. 2020). Beyond hearing loss (HL), noise triggers various psychological and physiological consequences, including tinnitus, fatigue, sleep disturbances, cognitive impairment, depression, anxiety, cardiovascular dysfunction, and immune system disorders (Mehrotra et al. 2024). Consequently, NIHL represents a major global health and social challenge, demanding comprehensive public strategies focused on prevention, early diagnosis and effective treatment.

Normal hearing requires a functional blood-labyrinth barrier at the stria vascularis (Nyberg et al. 2019) and precise intercellular communication within the organ of Corti in the cochlea (Glowatzki and Fuchs 2002). Sensory hair cells (HCs) perform mechano-electrical transduction via neurotransmitter release into synapses formed with spiral ganglion neurons (SGNs). Specifically, inner hair cells (IHCs) act as the primary mechanotransducers, functionally coupled to outer hair cells (OHCs) which enhance auditory sensitivity. Upon stimulation, IHCs quickly release the excitatory neurotransmitter glutamate that activates glutamate receptors (GluRs) on SGN afferent fibers. This rapid response relies on the clustering, anchoring and regulation of GluRs into large multiprotein complexes at the postsynaptic density (PSD). These complexes are organized by scaffolds proteins like PSD-95 and PSD-93, which interact with multiple partners including different types of GluRs [alpha-amino-3-hydroxy-5-methyl-4-isoxazole propionate receptors (AMPARs), N-methyl-D-aspartate receptors (NMDARs) and kainite receptors (KARs)] (Niedzielski and Wenthold 1995) as well as cytoskeleton, signaling and regulatory proteins (Davies et al. 2001). In addition to glutamate, efficient IHC-SGN communication also depends on the release by HCs and other cochlear cells of trophic factors, such as brain derived neurotrophic factor (BDNF) or neurotrophic factor 3 (NT-3) (Pirvola and Ylikoski 2003; Sugawara et al. 2007). These factors bind and activate their respective tyrosine kinase receptors, TrkB and TrkC, on SGNs, promoting neuronal survival, neurite growth, and synapse formation (Reichardt 2006). Although levels of these neurotrophins and their receptors are reduced after birth, they remain central to SGN maintenance in the adult inner ear (Frago et al. 2000; Green et al. 2012; Ma et al. 2019).

Excessive noise exposure can lead to either temporary or permanent HL depending on whether auditory thresholds fully recover or remain elevated over time. A key early event in temporary NIHL is the degeneration of IHC-SGN synaptic connections, a process that persists or worsens over time (Le et al. 2017; Kujawa and Liberman 2009) and precedes the loss of HCs and SGNs. Beyond NIHL, cochlear synaptopathy is also a critical pathological feature of age-related hearing loss (ARHL) (Sergeyenko et al. 2013). ARHL is not only the most common cause of deafness, affecting one-third of individuals over 65, but it also precedes and correlates with cognitive decline (Deal et al. 2017), representing a significant risk factor for dementia (Deal et al. 2017; Lin and Black 2017). Regardless of etiology, cochlear synaptopathy is a major contributor to “hidden hearing loss” (HHL), a condition characterized by normal audiometric thresholds but reduced amplitudes of sound-evoked auditory potentials (Kujawa and Liberman 2015), resulting in poor speech perception in noisy environments. Therefore, the rational development of therapeutic agents for HL requires a comprehensive understanding of the molecular mechanisms underlying cochlear synaptopathy.

Although several mechanisms are involved in the degeneration of cochlear synaptic connections, it is widely accepted that glutamate excitotoxicity plays a central role (Puel et al. 1998; Pujol et al. 1985; Hakuba et al. 2000; Kim et al. 2019). IHCs overstimulated by excessive noise release high glutamate concentrations to the synaptic cleft, causing overactivation of GluRs present at the SGN PSD and initiating excitotoxic damage (Kurabi et al. 2017). Excitotoxicity is mediated by massive Ca^2+^ entry in neurons (Hu et al. 2020) followed by stimulation of different enzymatic activities. These include, among others, the Ca^2+^-dependent protease calpain or enzymes producing free radicals, mitochondrial damage and subsequent induction of oxidative stress and proinflammatory processes (Saidia et al. 2024; Varela-Nieto et al. 2020; Murillo-Cuesta et al. 2025). In addition, evidence suggests that, in the cochlea, excitotoxic damage can also compromise neurotrophic support (Meltser et al. 2010; Saidia et al. 2024). A particular mechanism that might contribute to impaired neurotrophic signaling in excitotoxic conditions is the downregulation of the high affinity BDNF receptor isoform, TrkB full-length (TrkB-FL). Dysregulation of this receptor has been implicated in other excitotoxicity-associated pathologies, including neurodegenerative diseases (Alzheimer’s or Parkinson’s), neuropsychiatric disorders (depression or schizophrenia), and neurological conditions (epilepsy or stroke) (Tejeda and Diaz-Guerra 2017). In fact, TrkB-FL processing has been well-characterized in ischemic stroke and in vitro models of excitotoxic injury (Vidaurre et al. 2012; Tejeda et al. 2016), where receptor cleavage takes place in the Golgi apparatus due to calpain activation in a process secondary to TrkB-FL endocytosis and retrograde transport to this organelle (Tejeda et al. 2019; Esteban-Ortega et al. 2025). Furthermore, a central role of TrkB-FL has been demonstrated in the stability of the Golgi complex, the receptor recruitment to this organelle preceding and participating in its fragmentation (Esteban-Ortega et al. 2025). This is important because disruption on the Golgi apparatus is considered as a pathological hallmark common to different neurodegenerative disorders (Gonatas et al. 2006; Martinez-Menarguez et al. 2019). Downregulation of the TrkB-FL receptor compromises BDNF-mediated signaling and decreases the promoter activity of prosurvival transcription factors, such as cAMP response-element binding protein (CREB) (Bonni et al. 1999) and myocyte enhancer factor 2 (MEF2) (Liu et al. 2003; Wang et al. 2007), causing transcriptional changes that favor neuronal death (Tejeda et al. 2019).

Repairing cochlear synaptopathy is feasible during the extended therapeutic window while HCs and SGNs, irreplaceable postmitotic cells, still survive (Kujawa and Liberman 2009). Treatments with BDNF and NT-3 have shown neuroprotective potential on cochlear innervation and synapses in models of ex vivo excitotoxicity, based on cochlear explants (Szobota et al. 2019; Wang and Green 2011), or animal models of noise overexposure, where they improved hearing function (Wan et al. 2014; Suzuki et al. 2016; Sly et al. 2016; Cunningham and Tucci 2015). Additionally, therapeutic molecules targeting TrkB and TrkC receptors such as small-molecule agonists (Kempfle et al. 2021; Yu et al. 2013), monoclonal antibodies (Szobota et al. 2019; Vink et al. 2023) or chimeric neurotrophins (Szobota et al. 2019) have been also evaluated. In humans, neurotrophin-based therapy has proved to be more challenging. A phase 1/2 clinical trial involving intratympanic administration of a BDNF formulation in a thermo-reversible polymer to patients facing speech discrimination difficulties in noisy environments has provided limited evidence of improvement (Foster et al. 2022). Two critical limitations challenge efficient delivery and function of neurotrophins, or their analogs, in NIHL therapy. First, a combination of factors affecting inner ear accessibility, including cochlear encapsulation in one of the hardest body bones, impermeability of ear membranes (eardrum, round window and oval widow) and presence of the blood-labyrinth barrier separating the vasculature and inner ear fluids (Nyberg et al. 2019). Secondly, the predictable downregulation of the BDNF receptor TrkB-FL in the cochlea by noise-induced excitotoxic damage. However, TrkB-FL degradation can be prevented and BDNF-signaling preserved in conditions of excitotoxic injury using a rationally designed cell-penetrating peptide (CPP), MTFL_457_ (Tejeda et al. 2019). This peptide has two elements: a HIV-1 Tat basic domain, which confers membrane permeability and the capability of crossing the blood-brain barrier to attached cargoes (Kurrikoff and Langel 2019; Pirhaghi et al. 2024) and a short TrkB-FL sequence (aa 457–471) that interferes excitotoxicity-induced receptor retrograde transport to the Golgi apparatus, secondarily preventing TrkB-FL processing (Tejeda et al. 2019) and organelle fragmentation (Esteban-Ortega et al. 2025). The preserved receptor then triggers a feedback survival mechanism mediated by CREB and MEF2 promoter activities favoring expression of critical prosurvival mRNAs and proteins in neurons. Importantly, in a context of in vivo excitotoxicity produced by ischemic stroke, MTFL_457_ systemic administration also prevents TrkB-FL downregulation in the infarcted brain, efficiently decreases infarct size, and improves neurological outcome (Tejeda et al. 2019). Therefore, TrkB-FL unravels as a highly relevant target for treatment of excitotoxicity-associated pathologies, including NIHL and ARHL. This TrkB-FL-derived CPP might be particularly adequate as a therapy for these conditions since, similarly to the blood-brain barrier, it might be able to cross the blood-labyrinth barrier after systemic administration.

CPPs have attracted a significant interest as non-invasive therapeutic and drug-delivery systems for different nervous system diseases (Pirhaghi et al. 2024). They are generally short (4–40 aa) and positively charged peptides that can translocate across cellular membranes and biological barriers by different mechanisms (Kurrikoff and Langel 2019). Additionally, CPPs have a large capacity to carry different conjugated bioactive molecules, and low toxicity (Pirhaghi et al. 2024; Varnamkhasti et al. 2020). The results obtained in clinical trials with a CPP that blocks the c-Jun N-terminal kinase pathway (EudraCT 2013-002077-21) have provided a proof-of-concept that HL therapy with CPPs might be feasible (Staecker et al. 2019).

In this work, we hypothesize that MTFL_457_ might be able to cross the blood-labyrinth barrier after systemic administration, reach the cochlea and help to prevent synaptic injury induced by noise overexposure. To test MTFL_457_ effects in the inner ear, we employed an ex vivo model of excitotoxicity, where this process is induced in cochlear explants by treatment with GluRs agonists, and an in vivo model of noise overexposure. The results presented here are highly relevant since they provide further insights about the effects of excitotoxicity in cochlear synaptopathy, emphasizing the contribution of the dysregulation of TrkB-FL and downstream prosurvival signaling. These data also demonstrate the MTFL_457_ is actually able to cross the blood-labyrinth barrier and distribute through the cochlea, interfering TrkB-FL downregulation and being neuroprotective against ex vivo and in vivo excitotoxicity. The observation that MTFL_457_ preserves cochlear synapses and improves hearing function in a mice model of noise overexposure strongly supports the therapeutic potential of this peptide for treatment of NIHL and, probably, other types of HL similarly associated with excitotoxicity.

## Materials and methods

Antibodies, CPPs, chemicals, culture products, experimental models, software, and other resources are detailed in Supplementary Table-S1, which also specifies antibody dilutions and the working concentrations for each reagent.

### Experimental models

For ex vivo experiments, pregnant RjOrl: SWISS mice (15–16 days gestation) were obtained from Janvier Laboratories (France). Each female produced litters of 10–12 pups, and experiments were performed on two-day-old (P2) neonates without sex distinction. For in vivo studies, 2-month-old male and female C57BL/6J mice were obtained from the Instituto de Investigaciones Biomédicas Sols-Morreale (IIBM, Madrid, Spain), with experiments conducted separately by sex. All animals were housed at IIBM facilities (Comunidad de Madrid approval #ES 280790000188) under standard conditions: individually ventilated cages with bedding and nesting material, 12 h light/dark cycle, controlled temperature and humidity, and *ad libitum* access to irradiated food and water. Pregnant females were housed individually; adult mice in same-sex groups (< 6 per cage). All procedures complied with European (2010/63/EU) and Spanish (RD 53/2013) regulations and were approved by the CSIC Ethics Committee.

### Cochlear explant culture

Cochleae from P2 RjOrl: SWISS mice were dissected in ice-cold PBS, as previously described (Wang and Green 2011). Briefly, the membranous labyrinth was separated from the modiolus, and the stria vascularis and spiral ligament were carefully removed. The organ of Corti and spiral ganglion were maintained intact, while Reissner’s and tectorial membranes were removed using fine forceps. Explants were placed on cell culture inserts in 6-well plates with 2 ml per well of Dulbecco’s modified Eagle’s medium/nutrient mixture F-12 (DMEM/F-12) supplemented with 2 mM L-glutamine, N-2 supplement, insulin-transferrin-selenium, 8.25 mM D-glucose and 30 U/ml penicillin G. Explants were incubated at 37 °C in 5% CO₂ and were left untreated for 48 h post-dissection to allow adhesion.

### Induction of excitotoxicity and CPP treatment of cochlear explants

To evaluate excitotoxic damage, cochlear explants were treated for 2 h with different GluR agonists: NMDA and its co-activator glycine (herein denoted as NG), kainate (herein denoted as K), or a combination of both (herein denoted as NGK), as previously described (Tejeda et al. 2019; Wang and Green 2011). Following these treatments, explants were transferred to fresh culture medium and subsequently fixed with 4% paraformaldehyde in PBS at 2, 24 or 48 h after induction of excitotoxicity. Following preliminary experiments, NGK treatment was selected as the ex vivo excitotoxicity model used to evaluate the effects of CPPs on cochlear damage. Before of or immediately after 2 h of NGK treatment, cochlear explants were incubated for 6 h with Tat-derived peptides, MTMyc or MTFL_457_ (25 µM; >95% purity; GenScript). Acetylation and amidation of these peptides, respectively at their N-ter and C-ter, is directed to enhance their plasma stability. After that, explants were transferred to fresh culture medium and fixed 24 h after the induction of excitotoxicity. As control experimental conditions, explants treated only with NGK or CPPs were also included.

To evaluate CPP penetration and distribution, explants were incubated for 2, 6 and 24 h with biotin-conjugated peptides, Bio-MTMyc and Bio-MTFL_457_ (25 µM; >95% purity; GenScript). Substitution of the N-ter acetyl group of these peptides by a biotin moiety enabled their visualization in the explants.

Untreated explants, used as additional controls, were incubated in fresh culture medium and processed concurrently with the experimental cultures.

### In vivo CPP administration

For in vivo experiments, peptides were prepared as 2.5 mM solutions in 0.9% NaCl (saline), and acidity due to the trifluoracetic acid counterions present in this solution was neutralized by adding fresh HCO_3_NH_4_ to a final concentration of 44 mM. Possible adverse effects on auditory function, HC viability or cochlear synapses were evaluated in male mice i.p. injected with MTMyc or MTFL_457_ (10 nmol/g). Once confirmed that these CPPs do not significantly alter these parameters, we analyzed their effects in a model of NIHL. Therefore, male and female mice were i.p. injected with MTFL_457_ (or MTMyc for male mice) (10 nmol/g) 2 h before noise exposure. Hearing function was evaluated by the ABR test before (baseline) and 1 day after CPP administration or after CPP pre-treatment and noise exposure as indicated. Animals were then sacrificed and cochleae were collected. Control animals (untreated and non-exposed) and animals subjected exclusively to noise exposure were included in these experiments.

### Hearing evaluation

ABRs were recorded using a Tucker David Technologies workstation (TDT, Tucker Davis Technologies), as previously reported (Cediel et al. 2006). Mice were anesthetized by i.p. injection with ketamine and xylazine, and recordings were performed in a sound-attenuating chamber. Two different sound stimuli, broadband click and tone-bursts (8, 16, 24, 32 and 40 kHz), were generated using SigGenRP™ software (Tucker-Davis Technologies) and presented monaurally at a decreasing intensity rate from 90 to 20 dB SPL in 5–10 dB steps. The electroencephalographic activity was recorded using stainless steel needle electrodes (TE/S50716-001; Technomed), and subsequently amplified and averaged. ABR thresholds were determined by visual detection and defined as the lowest intensity to elicit a reliable ABR wave with peaks I to IV clearly visible. Wave I amplitudes in response to click and tone-burst (24 and 32 kHz) stimuli were also analyzed.

### Noise exposure

To assess noise susceptibility, conscious mice were placed in a wire-mesh cage at the center of a reverberant chamber designed for uniform sound distribution (Cobo et al. 2009) and exposed to violet swept-sine noise (VSS) (105 dB SPL, 30 min) (Partearroyo et al. 2019; Sanz et al. 2015). Male and female experiments were conducted separately. The stimulus, generated with Wavelab Lite (Steinberg Media Technologies GmbH), consisted of 10 s linear frequency sweeps (2–20 kHz) with frequency-dependent gain to compensate for high-frequency attenuation. Hearing function after exposure was evaluated by ABR testing as described above.

### Peptide visualization in cochlear explants

Cochlear explants incubated with Bio-MTMyc, Bio-MTFL_457_ or left untreated, as above indicated, were subsequently fixed in 4% PFA in PBS. Following three washes with PBS, explants were permeabilized and blocked with 0.3% Triton X-100 and 30% goat serum in PBS for 30 min at room temperature. Then, they were incubated overnight at 4 °C with fluorescein (FITC)-avidin D diluted in 0.1% Triton X-100 and 1% goat serum in PBS. After three additional PBS washes, explants were mounted in Fluoromount-G. High magnification (63x) fluorescent z-stack images were acquired using a Zeiss LSM710 confocal laser-scanning microscope, and qualitative evaluation of peptide presence was performed in the organ of Corti and spiral ganglion. A minimum of three explants per experimental condition were used.

### Peptide visualization in treated animals

The ability of CPPs to access and distribute throughout the inner ear was assessed by i.p. injecting male with Bio-MTMyc/Bio-MTFL_457_ (10 nmol/g, prepared as described above) or vehicle (saline). Animals were sacrificed at 30 min–2 h after peptide administration with a pentobarbital overdose and intracardially perfused with cold PBS and 4% PFA in PBS. Dissected cochleae were post-fixed with 4% PFA, decalcified in 5% EDTA, and embedded in Tissue-Tek OCT. Subsequently, frozen OCT cochlear adherent Sect.  (10 μm) were obtained using a Cryocut 1950 (Leica Microsystems) and were permeabilized and blocked with 0.5% Triton X-100 and 10% goat serum in PBS for 2 h at room temperature. After that, sections were incubated with FITC-avidin D in 4% goat serum in PBS overnight at 4 °C. Following three PBS washes, sections were stained with TO-PRO3 for 10 min at room temperature to label cell nuclei. Finally, after three additional PBS washes, sections were mounted in Fluoromount-G. High magnification (63x) fluorescent z-stack images of the organ of Corti, spiral ganglion and stria vascularis in each cochlear turn were acquired as above indicated. Peptide detection was qualitatively analyzed in 2–4 equivalent cryosections per animal from at least two mice per condition.

### Immunofluorescence and labeling of degenerating SGNs in cochlear explants

All procedures were performed with cochlear explants which remained attached to the culture insert membrane. After fixation and washing with PBS, unless otherwise indicated, explants were permeabilized and blocked with 0.3% Triton X-100 and 30% goat serum in PBS for 30 min at room temperature. Explants were then sequentially incubated with primary and secondary antibodies diluted as indicated (Supplementary Table-S1) in 0.1% Triton X-100 and 1% goat serum in PBS. Incubation with primary antibodies was overnight at 4 °C and that with secondary antibodies was for 2 h at room temperature, both separated by three PBS washes. Primary antibodies included anti-NF200, which labels SGNs and their peripheral axons, anti-GM130, for detection of the *cis*-Golgi, anti-TrkB-FL, to specifically label this TrkB isoform, and anti-p-CREB, for detection of Ser133 phosphorylated CREB (p-CREB), generally considered as the active form of this transcription factor. Phalloidin-647 was co-incubated with primary antibodies to label HC apical structures. Secondary antibodies were goat Alexa Fluor conjugates 488 and 546. Finally, after three PBS washes, explants were mounted in Fluoromount-G.

HCs and cochlear synapses were immunolabeled following a modified protocol (Liberman et al. 2014). After fixation and washing with PBS, the explants were permeabilized and blocked with 1% Triton X-100 and 7% goat serum in PBS for 1 h at room temperature. Primary and secondary antibodies were diluted as indicated (Supplementary Table-S1) in 0.25% Triton X-100 and 1% goat serum in PBS. Incubation with the primary antibodies proceeded for 16–19 h at 37 °C, and that with secondary antibodies for 2 h at room temperature, both separated by three PBS washes. Primary antibodies included anti-MyoVIIa, for HC labelling, anti-CtBP2 (IgG_1_) to label HC ribbon synapses (presynaptic puncta), and anti-PSD-95 (IgG_2a_) to label PSDs (postsynaptic puncta). Secondary antibodies were goat anti-rabbit Pacific Blue for MyoVIIa detection, and IgG subclass-specific antibodies for CTBP2/PSD-95 double labeling: respectively, goat anti-mouse IgG1 Alexa Fluor 488 and goat anti-mouse IgG2a Alexa Fluor 555. Following three PBS washes, explants were stained with TO-PRO3 for 30 min at room temperature to label cell nuclei. Finally, after three additional PBS washes, explants were mounted as previously described.

Specific labeling of degenerating SGNs was performed using FJC staining. Following secondary antibodies incubation and a distilled water wash, explants were incubated with a solution of 0.0004% FJC in 0.01% acetic acid for 10 min at room temperature, and then mounted as described above.

### Organ of Corti whole mount dissection and immunofluorescence

Whole mount dissection of the Organ of Corti was performed as previously described (Bermudez-Munoz et al. 2020; Montgomery and Cox 2016) in animals euthanized with a pentobarbital (Dolethal; Vetoquinol) overdose and intracardially perfused with cold PBS and 4% PFA in PBS. Sampled cochleae were post-fixed in 4% PFA and decalcified in 5% EDTA, followed by cochleae dissection into five half-turns, from apex to base, carefully removing stria vascularis and Reissner’s and tectorial membranes, while preserving the organ of Corti and SGN afferent innervation. Then, the organ of Corti pieces were transferred to 72-well plates for free-floating immunofluorescence. The protocol for immunolabeling of HCs and cochlear synapses was as described for cochlear explants (Liberman et al. 2014), except that an anti-GluR2 antibody (IgG_2a_) was used as the postsynaptic marker.

### Qualitative evaluation of the integrity of SGN afferent innervation

High magnification (63x) fluorescent z-stack images of SGN afferent innervation within the middle turn of cochlear explants were acquired with a Zeiss LSM710 confocal laser-scanning microscope. The structural integrity of the afferent innervation was qualitatively assessed by examining changes in fiber density, as well as the swelling or loss of synaptic terminals. At least 3 explants per condition were evaluated.

### Quantitative analysis of immunofluorescence intensity

High magnification (63x) fluorescent z-stack images of the organ of Corti and the spiral ganglion within the middle turn of cochlear explants were acquired as above indicated. Total staining intensity for TrkB-FL, p-CREB and FJC was quantified using custom macros in Fiji software (Schindelin et al. 2012). Immunofluorescence intensity for each marker was normalized to their respective untreated explants (assigned an arbitrary value of 100% for TrkB-FL and p-CREB, or 1 for FJC). FJC primarily labels the cell body of degenerating neurons (Tejeda et al. 2016).

### Evaluation of Golgi apparatus fragmentation

To assess fragmentation of SGN Golgi complex within the middle turn of cochlear explants, high magnification (63x) fluorescent z-stack images were acquired as before, and 3D reconstructions were generated using IMARIS (Bitplane Inc.), as previously described (Thayer et al. 2013). Individual regions of interest (ROI) for the *cis*-Golgi (GM130) within the SGNs (NF200) were defined in each 3D image using an empirically established maximum sphere diameter of 0.988 μm, which was followed by background subtraction function, and manual threshold adjustment to capture all relevant elements. The total number of Golgi fragments and their volume were quantified and normalized to the number of SGNs analyzed in each experimental condition (*n* = 49–60 neurons, from at least 3 explants per condition). An intact Golgi corresponds to fewer, larger structures, whereas a fragmented Golgi is characterized by more numerous, smaller structures.

### HC and cochlear synapse quantification

For HC quantification, low magnification (25x) fluorescent images of both cochlear explants and organ of Corti whole mounts were captured as before indicated, the latter being used for cochleogram analysis with a Fiji plugin (Bermudez-Munoz et al. 2020; Liberman et al. 2014). IHCs and OHCs were counted in a 200 μm segment of the basilar membrane within the middle turn of cochlear explants or, in the case of organ of Corti whole mounts, in the 8 (apical), 16–24 (middle) and 32 (basal) kHz regions.

For quantification of cochlear synapses, high magnification (63x) fluorescent z-stack images were acquired as before, and 3D reconstructions were generated using IMARIS (Bitplane Inc.), as previously described (Bermudez-Munoz et al. 2020). The number of colocalized presynaptic ribbons (CtBP2 puncta) and PSDs (PSD-95 puncta) or GluRs patches (GluR2 puncta) per IHC was quantified in the cochlear regions above specified, both for cochlear explants and organ of Corti whole mounts. In addition, the total number of puncta for each individual marker per IHC was determined. For each marker (CtBP2 and PSD-95/GluR2), spots were generated in independent channels using an XY diameter of 1 μm and a corrected Z diameter point spread function (PSF) of 1.5 μm. The background subtraction function was activated and the intensity threshold was manually adjusted to capture all elements of interest. Spot colocalization measurements were used to analyze pairing of presynaptic and postsynaptic elements, using a 1 μm maximum distance threshold to define juxtaposition of two different puncta. The computed results were corroborated by visual inspection of the puncta.

### Statistical analysis

Except for violin plots, data are expressed as mean ± standard error of the mean (SEM). Statistical analysis was performed in GraphPad Prism 8.0.2. Normality of the data was assessed by using the Shapiro-Wilk test. The number of explants or animals included in each experiment, as well as the specific statistical test applied, are detailed in the corresponding figure legends. In all cases P-value significance is considered as: * *P* < 0.05, ** *P* < 0.01, *** *P* < 0.001, **** *P* < 0.0001. P-values larger than 0.05 were considered non-significant (n.s.).

## Results

### Excitotoxicity induces neurodegeneration and synaptopathy in cochlear explants with no effect on HCs viability

**Fig. 1:**
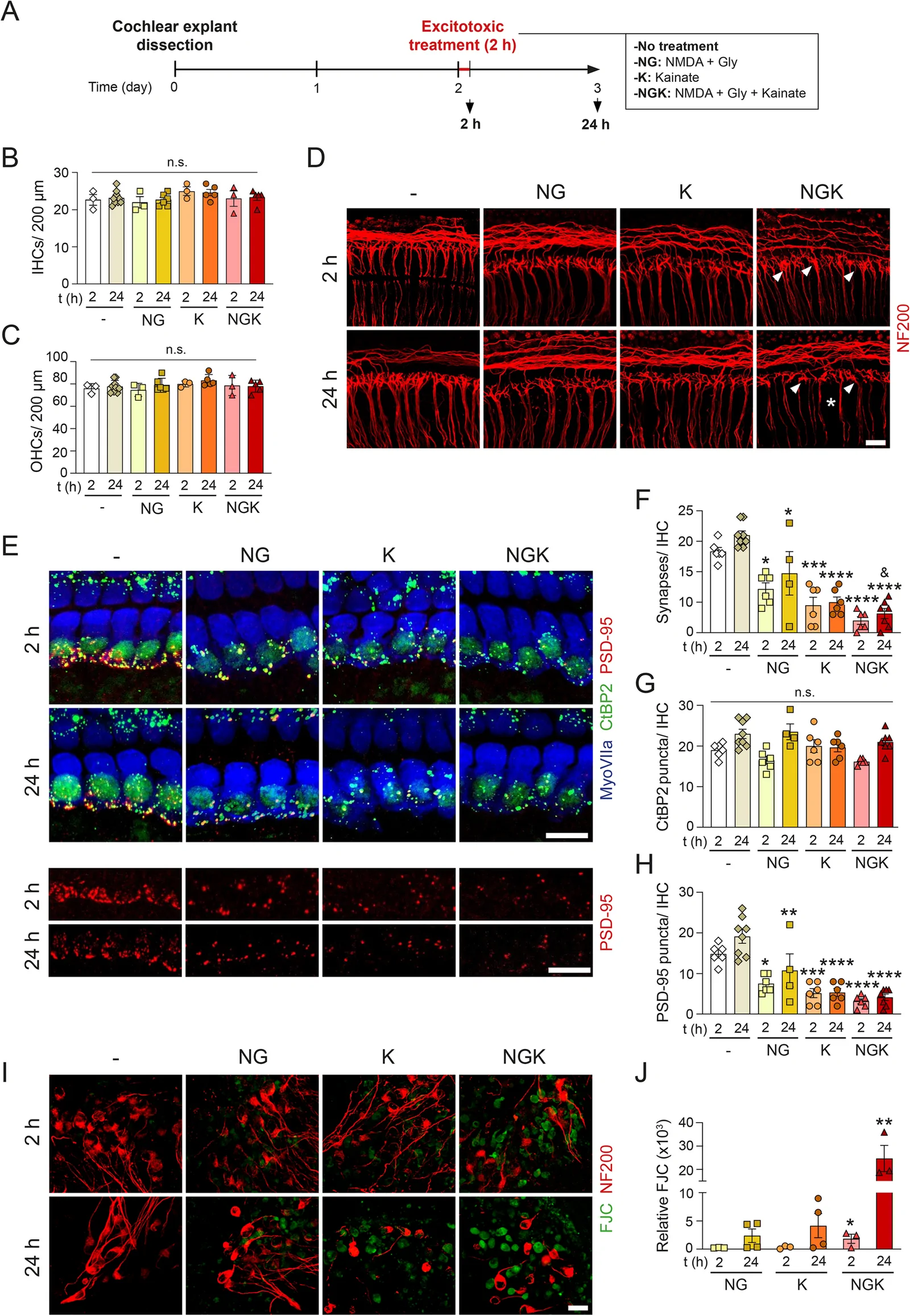
Excitotoxicity induces neurodegeneration and synaptopathy in cochlear explants with no effect on HCs viability. **A** Experimental design. Cochlear explants, 2 days post-dissection, were treated for 2 h with different GluR agonists: NG (NMDA, 500 µM, and glycine, 10 µM), K (kainate, 500 µM) or NGK (a combination of both treatments). Subsequently, explants were fixed 2 or 24 h after excitotoxic damage induction and processed for immunofluorescence, focusing on the cochlear middle turn. **B-C** Quantification of IHCs (**B**) and OHCs (**C**) over a 200 μm segment of the basilar membrane after the different excitotoxic treatments. Means ± SEM are represented (*n* = 3–9 explants/condition). Statistical analysis was assessed by Kruskal-Wallis test followed by Dunn’s multiple comparison test: n.s., non-significant. **D** Representative confocal images showing the progressive degeneration of synaptic terminals and afferent fibers of SGNs, labeled for neurofilament 200 kDa (NF200, red), following excitotoxic damage. Asterisk and arrowheads in NGK-treated explants indicate, respectively, the absence or presence of fibers and synaptic terminals. Qualitative analysis of the structural integrity of cochlear afferent innervation was performed using 3–4 explants per experimental condition. Scale bar: 20 μm. **E** Representative confocal images showing the impact of excitotoxicity on IHC cochlear synapses labeled with antibodies for MyoVIIa (HC marker, blue), CtBP2 (presynaptic marker, green), and PSD-95 (postsynaptic marker, red). The upper panel shows IHC cochlear synapses (colocalized CtBP2/PSD-95 puncta), whereas the lower panel highlights only the postsynaptic component (PSD-95). Scale bars: 10 μm. (F-H) Number of synapses (**F**) and total CtBP2 (**G**) and PSD-95 (**H**) puncta per IHC. Means ± SEM are represented (*n* = 4–8 explants/condition). Statistical analysis was performed by one-way ANOVA test followed by Tukey’s multiple comparison test: * NG/K/NGK 2/24 h vs. respective controls, & NGK 24 h vs. NG 24 h (*, & *P* < 0.05; ** *P* < 0.01; *** *P* < 0.001; **** *P* < 0.0001). **I** Representative confocal images of spiral ganglion neurodegenerative damage under different excitotoxic conditions. Explants were stained with a specific neurodegeneration marker, Fluoro-Jade C (FJC, green), and labeled for NF200 (red). Scale bar: 20 μm. **J** Analysis of FJC marker levels, relative to their respective controls (untreated explants, assigned an arbitrary value of 1). Data are presented as mean ± SEM (*n* = 3–4 explants/condition). Statistically significant differences were established using Kruskal-Wallis test followed by Dunn’s multiple comparison test: * NGK 2/24 h vs. respective controls (* *P* < 0.05; ** *P* < 0.01)

Previous studies have shown that excessive glutamate release and the subsequent overactivation of GluRs lead to excitotoxic damage in the cochlea (Puel et al. 1991, 1994; Pujol et al. 1985). To establish a suitable ex vivo model of excitotoxicity for this study, we prepared cochlear explants from P2 mice, which preserve the integrity of the organ of Corti, the spiral ganglion, and the characteristic cochlear architecture (Supplementary Fig. 1). Excitotoxicity was induced in these explants by a 2 h treatment with different pharmacological glutamate analogs: NG (NMDA, 500 µM, combined with its co-activator glycine, 10 µM, specifically targeting NMDAR-type GluRs), K (kainate, 500 µM, activating KARs and AMPARs), or NGK (a combination of NG and K). The effects of these GluR agonists were assessed in the cochlear middle turn at 2 and 24 h post-treatment (Fig. 1A) and, occasionally, 48 h (Supplementary Fig. 2A-G). Overactivation of GluRs did not compromise HC viability, as no significant differences were observed in the number of IHCs (Fig. 1B) or OHCs (Fig. 1C). However, glutamate-induced excitotoxicity caused marked disruption of the structural integrity of type I SGN afferent fibers, visualized by neurofilament 200 kDa (NF200) immunolabeling. Pathological changes, including swelling, synaptic terminal loss, and afferent fiber retraction, were evident early after treatment and became particularly pronounced following 24 h (Fig. 1D) and 48 h (Supplementary Fig. 2B) of NGK exposure.

Next, we assessed the impact of these excitotoxic treatments on afferent cochlear synapses in IHCs using triple immunolabeling for MyoVIIa (HCs), CtBP2 (presynaptic ribbons), and PSD-95 (PSDs) (Fig. 1E), followed by colocalization analysis of pre- and postsynaptic markers. All excitotoxic treatments significantly decreased the number of synapses per IHC compared to untreated explants; however, synaptopathy was most severe after treatment with K and NGK, causing decreases of respectively 60–70% and 80–85% (Fig. 1F). These findings indicate that synaptic loss primarily results from destabilization of postsynaptic terminals, as the total number of presynaptic CtBP2 puncta per IHC remained unchanged (Fig. 1G), suggesting largely intact ribbon structures. In contrast, the number of postsynaptic PSD-95 puncta per IHC was significantly reduced (Fig. 1H). Moreover, neither synapse counts nor PSD-95 puncta differed significantly between 2 and 24 h for any treatment, suggesting that synaptic loss is an early event preceding afferent fiber degeneration.

Excitotoxicity not only induced the degeneration of cochlear innervation but also impacted the SGN somata. Using Fluoro-Jade C (FJC), a specific marker of neurodegeneration, we observed time-dependent neuronal degeneration in the spiral ganglion for all the different glutamate analogs analyzed (Fig. 1I). Treatments with NG and K induced similar increases in FJC levels, reaching their maximal values at 48 h (Supplementary Fig. 2C-D). Neurodegeneration further increased in NGK-treated explants, values being significantly higher compared to their respective untreated controls (respectively, 2,000-fold, 25,000-fold and 130,000-fold increases for 2, 24 and 48 h of damage; Fig. 1J and Supplementary Fig. 2D).

Altogether, these findings indicate that the excitotoxic damage induced by the combined application of the different glutamate analogs is more severe than that caused by individual GluR agonists, likely due to the simultaneous overactivation of NMDARs, AMPARs and KARs. Moreover, this combined activation of the different types of GluRs would more closely mimic the in vivo situation where excitotoxicity is induced by glutamate. Based on these results, we selected NGK treatment as our ex vivo excitotoxicity model for subsequent experiments.

### Peptide MTFL_**457**_ is neuroprotective in ex vivo excitotoxicity

Peptide MTFL_457_, a Tat-derived CPP containing a short sequence of TrkB-FL receptor (Supplementary Fig. 3A), has been previously reported as having neuroprotective effects in models of excitotoxic damage induced by GluR agonists in primary cultures of cortical neurons and models of cerebral ischemia (Tejeda et al. 2019). In those experiments, a similar Tat-derived CPP containing a short unrelated c-Myc sequence (MTMyc) was employed as a negative control (Supplementary Fig. 3A). This peptide has no effects on neuronal viability in basal or excitotoxic conditions and therefore it does not confer functional or synaptic protection (Tejeda et al. 2019; Ayuso-Dolado et al. 2021). To investigate the possibility of these CPPs being able to penetrate and distribute throughout cochlear explants, we designed their biotin-labelled forms, respectively Bio-MTFL_457_ and Bio-MTMyc (Supplementary Fig. 3A). Then, we incubated cochlear explants with the biotinylated peptides (25 µM) for 2, 6 or 24 h and found that, compared to the untreated explants, they were detected in the middle turn of cochlear explants, both at the organ of Corti (Supplementary Fig. 3B) and the spiral ganglion (Supplementary Fig. 3C), at all tested times, although the signal seemed to be slightly higher after 6 h of incubation.

**Fig. 2:**
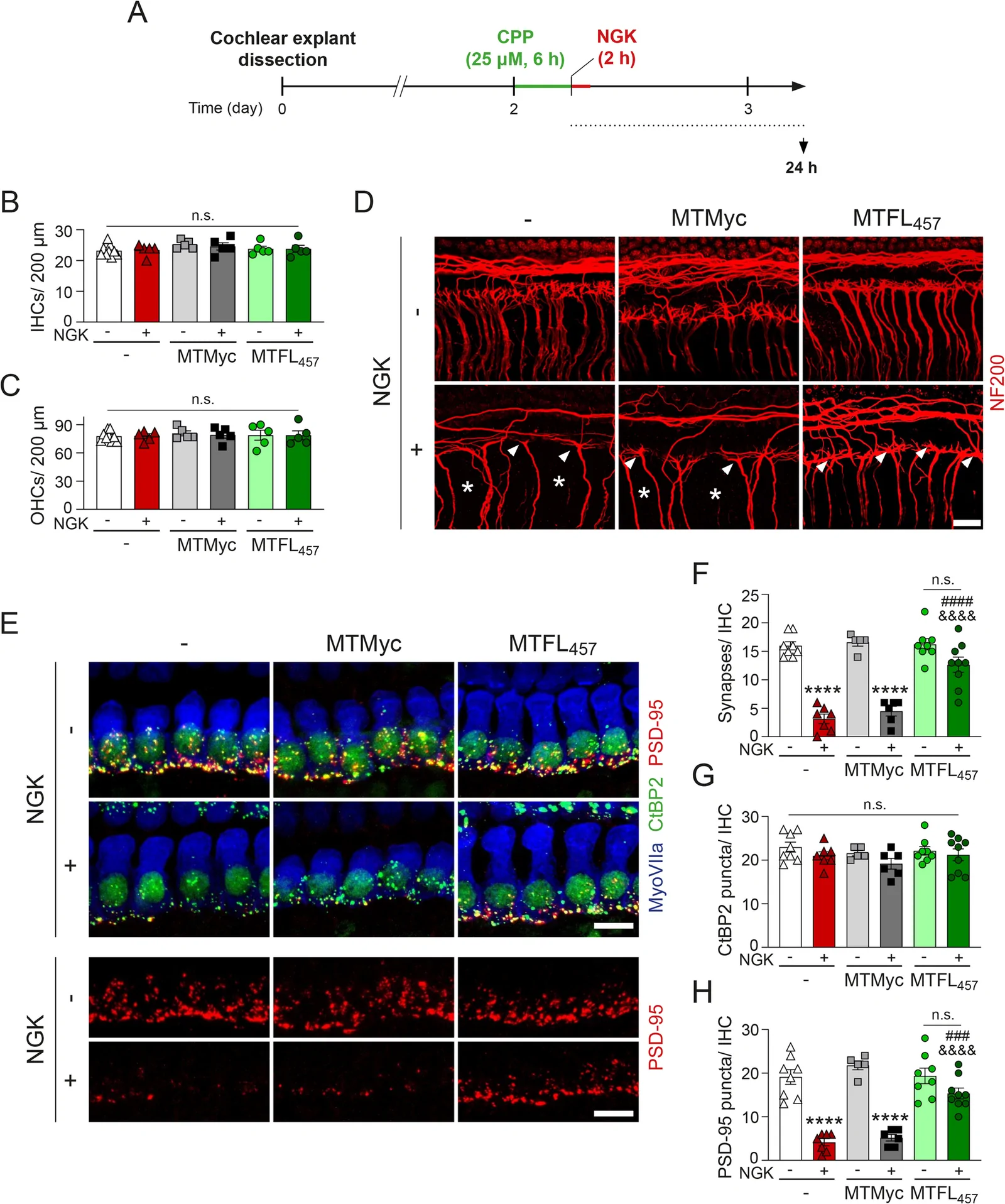
MTFL_457_ pre-treatment preserves SGN afferent fibers and cochlear synapses under excitotoxic conditions. **A** Experimental design. Cochlear explants, 2 days post-dissection, were pre-incubated with or without CPPs, MTFL_457_ or MTMyc (25 µM, 6 h), and then treated for 2 h with NGK or left untreated. Later on, 24 h after the induction of excitotoxicity, explants were fixed and processed for immunofluorescence, focusing on the cochlear middle turn. **B-C** Quantification of IHCs (**B**) and OHCs (**C**) over a 200 μm segment of the basilar membrane following CPP pre-treatment and excitotoxic damage. Data are presented as mean ± SEM (*n* = 5–9 explants/condition). Statistical analysis was assessed by Kruskal-Wallis test followed by Dunn’s multiple comparison test (**B**) or one-way ANOVA test followed by Tukey’s multiple comparison test (**C**): n.s., non-significant. **D** Representative confocal images illustrating the effect of MTFL_457_ on SGN synaptic terminals and afferent fibers, labeled with antibodies for NF200 (red), after excitotoxic injury. Asterisks and arrowheads denote, respectively, the absence or presence of fibers and synaptic terminals. Qualitative analysis of the structural integrity of cochlear afferent innervation was performed using 3–4 explants per experimental condition. Scale bar: 20 μm. **E** Representative confocal images showing the impact of MTFL_457_ on cochlear synaptopathy after excitotoxic damage. Explants were labeled with antibodies for MyoVIIa (blue), CtBP2 (green), and PSD-95 (red). The upper panel shows IHC cochlear synapses (colocalized CtBP2/PSD-95 puncta), whereas the lower panel highlights only the postsynaptic component (PSD-95). Scale bars: 10 μm. (F-H) Quantification of synapses (**F**) and total CtBP2 (**G**) and PSD-95 (**H**) puncta per IHC. Means ± SEM are represented (*n* = 5–9 explants/condition). Statistical analysis was performed by one-way ANOVA test followed by Tukey’s multiple comparison test: * NGK/MTMyc-NGK vs. respective control, & MTFL_457_-NGK vs. NGK, # MTFL_457_-NGK vs. MTMyc-NGK (n.s., non-significant; ### *P* < 0.001; ****, &&&&, #### *P* < 0.0001)

Next, we investigated the neuroprotective potential of MTFL_457_ in the ex vivo model of excitotoxicity previously established. Thus, explants were pre-treated for 6 h with MTMyc or MTFL_457_ (25 µM), or left untreated, before induction of excitotoxicity with NGK for 2 h. The effects of these treatments on cochlear middle turn were assessed 24 h after the induction of damage (Fig. 2A). Neither peptide had an effect on the number of IHCs (Fig. 2B) or OHCs (Fig. 2C), independently of the induction of the excitotoxic damage. In contrast, pre-treatment with MTFL_457_ had a protective effect on cochlear afferent innervation, partially preserving the structure of Type I SGN fibers in explants subjected to excitotoxicity. The protective effect of MTFL_457_ was specific and could not be observed for MTMyc- pre-treated explants, presenting an afferent fiber degeneration similar to that observed in explants treated with NGK alone (Fig. 2D).

Cochlear synaptopathy induced by the excitotoxic treatment was also efficiently prevented by MTFL_457_. The number of synapses per IHC in MTFL_457_-pre-treated explants was significantly higher than in explants treated with MTMyc + NGK or NGK alone (13 ± 1 vs. 5 ± 1 and 3 ± 1, *P* < 0.0001, respectively). Moreover, for MTFL_457_, no significant differences were observed between explants treated or not with NGK while a significant synaptic loss was induced by NGK in explants treated with MTMyc or no peptide (Fig. 2E-F). Consistent with previous results, the total number of CtBP2 puncta per IHC did not differ across any of the experimental conditions (Fig. 2G). Notably, the protective effect of MTFL_457_ was followed by PSD stabilization, evidenced by the significant increase of the total number of PSD-95 puncta per IHC in MTFL_457_-pre-treated explants undergoing excitotoxicity compared to those treated with MTMyc + NGK or NGK alone (Fig. 2H).

**Fig. 3:**
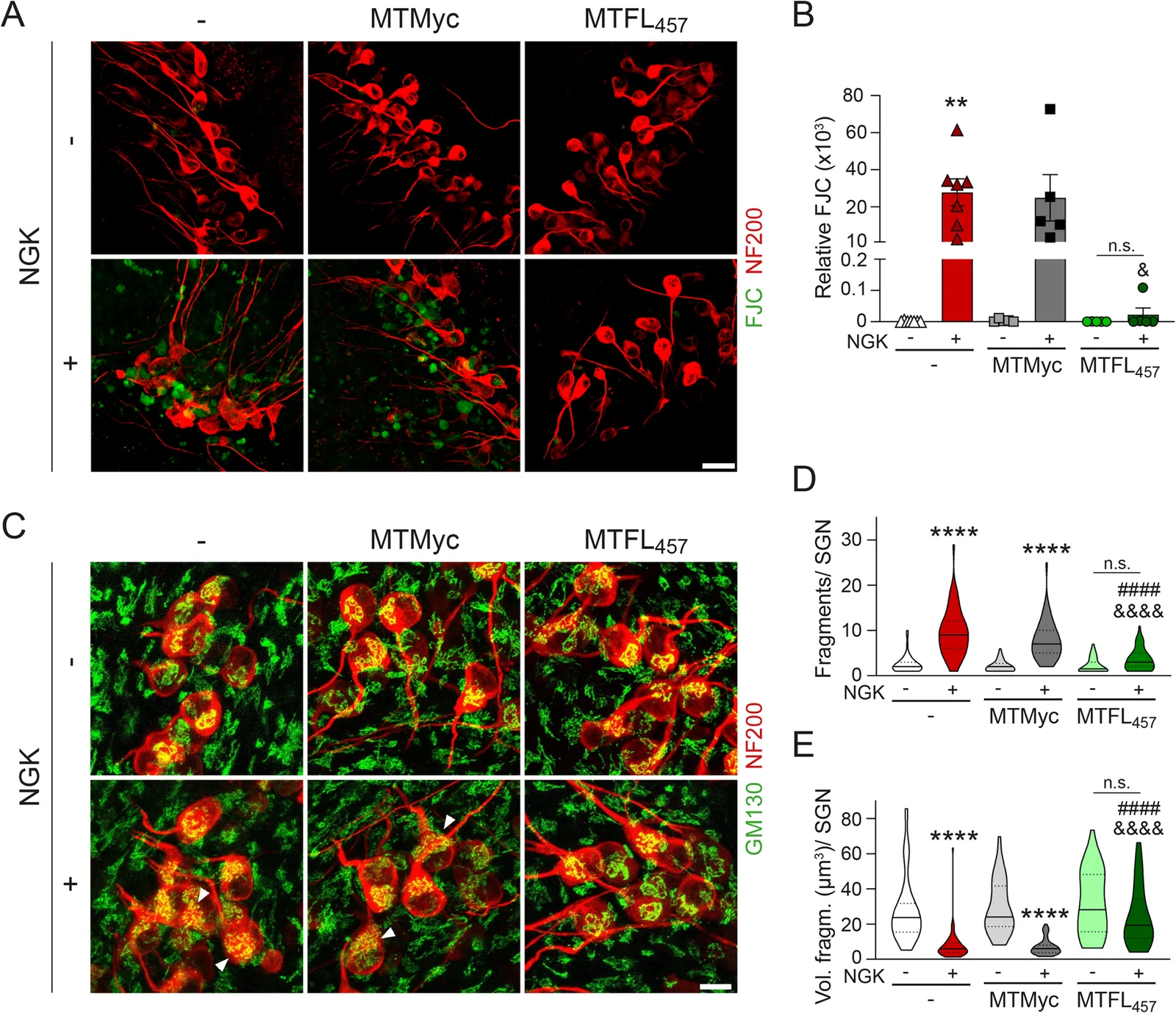
MTFL_457_ pre-treatment has a neuroprotective effect against excitotoxicity-induced spiral ganglion neurodegeneration. **A** Representative confocal images showing MTFL_457_ effect on spiral ganglion neurodegeneration induced by an excitotoxic insult. Cochlear explants were pre-incubated with MTFL_457_ or MTMyc (6 h), or no peptide, and then treated with NGK (2 h) as above indicated (Fig. 2A). As additional controls, the induction of excitotoxicity was omitted for some explants. After 24 h, explants were fixed and stained with FJC (green) and labeled for NF200 (red). Scale bar: 20 μm. **B** Quantification of FJC marker levels relative to untreated explants (assigned an arbitrary value of 1). Means ± SEM are represented (*n* = 5–7 explants/condition). Statistically significant differences were detected by Kruskal-Wallis test followed by Dunn’s multiple comparison test: * NGK vs. respective control, & MTFL_457_-NGK vs. NGK (n.s., non-significant; & *P* < 0.05; ** *P* < 0.01). **C** Representative confocal images showing excitotoxicity-induced Golgi fragmentation in SGNs and the effect of MTFL_457_. Explants, treated as previously described, were labeled with a specific *cis*-Golgi marker, GM130 (green), and NF200 (red). Fragmented organelles are indicated by arrowheads. Scale bar: 10 μm. **D-E** Quantification of the number (**D**) and volume (**E**) of Golgi fragments per SGN. Data are presented as mean ± quartiles (*n* = 49–60 neurons from at least 3 explants/condition). Statistical analysis was performed by Kruskal-Wallis test followed by Dunn’s multiple comparison test: NGK/ MTMyc-NGK vs. respective control, & MTFL_457_-NGK vs. NGK, # MTFL_457_-NGK vs. MTMyc-NGK (n.s., non-significant; ****, &&&&, #### *P* < 0.0001)

Then, we evaluated the effects of MTFL_457_ pre-treatment on additional indicators of spiral ganglion neurodegeneration induced by excitotoxicity. This peptide remarkably prevented the increase in FJC levels due to NGK treatment that was found in explants treated with MTMyc + NGK or NGK alone (Fig. 3A-B). Finally, we also investigated fragmentation of the Golgi apparatus in SGNs. Damage to this organelle is considered a characteristic marker of excitotoxicity-induced neurodegeneration, and is frequently observed at early stages of different neurodegenerative diseases (Martinez-Menarguez et al. 2019; Gonatas et al. 2006). Using the *cis*-Golgi specific marker, GM130, we observed a significant increase in the number of Golgi fragments in SGNs of explants treated with MTMyc + NGK or NGK alone (Fig. 3C-D), which corresponded with a significant reduction in fragment volume (Fig. 3E). Conversely, MTFL_457_ pre-treatment was able to preserve the structural integrity of the organelle, being the number and volume of the fragments in excitotoxic conditions comparable to those of the respective undamaged control (Fig. 3D-E).

**Fig. 4:**
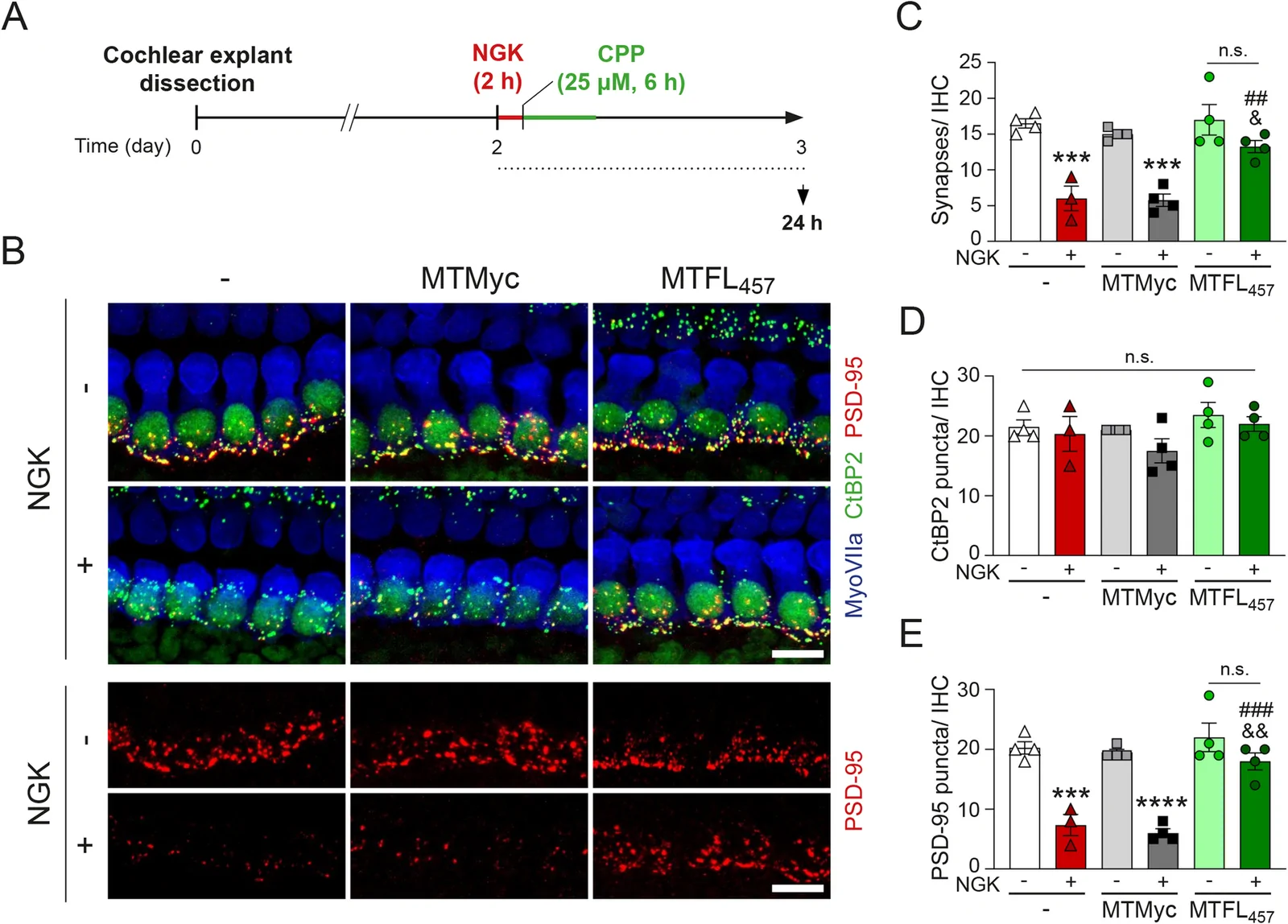
Administration of MTFL_457_ after the onset of the excitotoxic injury protects cochlear synapses. **A** Experimental design. Cochlear explants were treated with NGK (2 h), or left untreated, followed by incubation with MTFL_457_, MTMyc or no peptide (6 h). Then, explants were fixed 24 h after the induction of excitotoxic damage and processed for immunofluorescence, focusing on the middle turn. Samples were labeled with antibodies for MyoVIIa (blue), CtBP2 (green), and PSD-95 (red). **B** Representative confocal images showing the effect of MTFL_457_ post-treatment on cochlear synapses under excitotoxic conditions. The upper panel shows IHCs cochlear synapses (colocalized CtBP2/PSD-95 puncta), whereas the lower panel highlights only the postsynaptic component (PSD-95). Scale bars: 10 μm. **C-E** Quantification of synapses (**C**) and total CtBP2 (**D**) and PSD-95 (**E**) puncta per IHC. Data are presented as means ± SEM (*n* = 3–4 explants/condition). Statistical analysis was performed by one-way ANOVA test followed by Tukey’s multiple comparison test: * NGK/NGK-MTMyc vs. respective control, & NGK-MTFL_457_ vs. NGK, # NGK-MTFL_457_ vs. NGK-MTMyc (n.s., nonsignificant; & *P* < 0.05; &&, ## *P* < 0.01; ***, ### *P* < 0.001; **** *P* < 0.0001)

The neuroprotective efficacy of pre-treatment with MTFL_457_ encouraged us to investigate whether this peptide had similar efficiency when administered once the excitotoxic damage is already established. Thus, cochlear explants were first treated with NGK for 2 h and, after removing the glutamate analogs, incubation proceeded for 6 h with MTMyc or MTFL_457_ (25 µM) or no peptide. As before, cochlear synapses were then evaluated 24 h after excitotoxicity induction (Fig. 4A). Interestingly, the number of cochlear synapses per IHC in the MTFL_457_ post-treated explants subjected to excitotoxicity was significantly higher compared to those treated with MTMyc + NGK (13 ± 1 vs. 6 ± 1, *P* < 0.01) or NGK alone (13 ± 1 vs. 6 ± 2, *P* < 0.05) (Fig. 4B-C). As expected, there were no changes in the total number of CtBP2 puncta per IHC (Fig. 4D), although a significant increase in the total PSD-95 puncta per IHC was observed in excitotoxic conditions after MTFL_457_ treatment (Fig. 4E). Therefore, these results suggest that MTFL_457_ as a post-treatment has also a protective effect on cochlear synapses, contributing again to PSD stabilization. This result further supports the great potential of MTFL_457_ to prevent cochlear synaptopathy and neurodegeneration in excitotoxic conditions and its feasibility for clinical application.

### Neuroprotection by MTFL_**457**_ pre-treatment in ex vivo excitotoxicity derives from stabilization of TrkB-FL receptor and downstream signaling

**Fig. 5:**
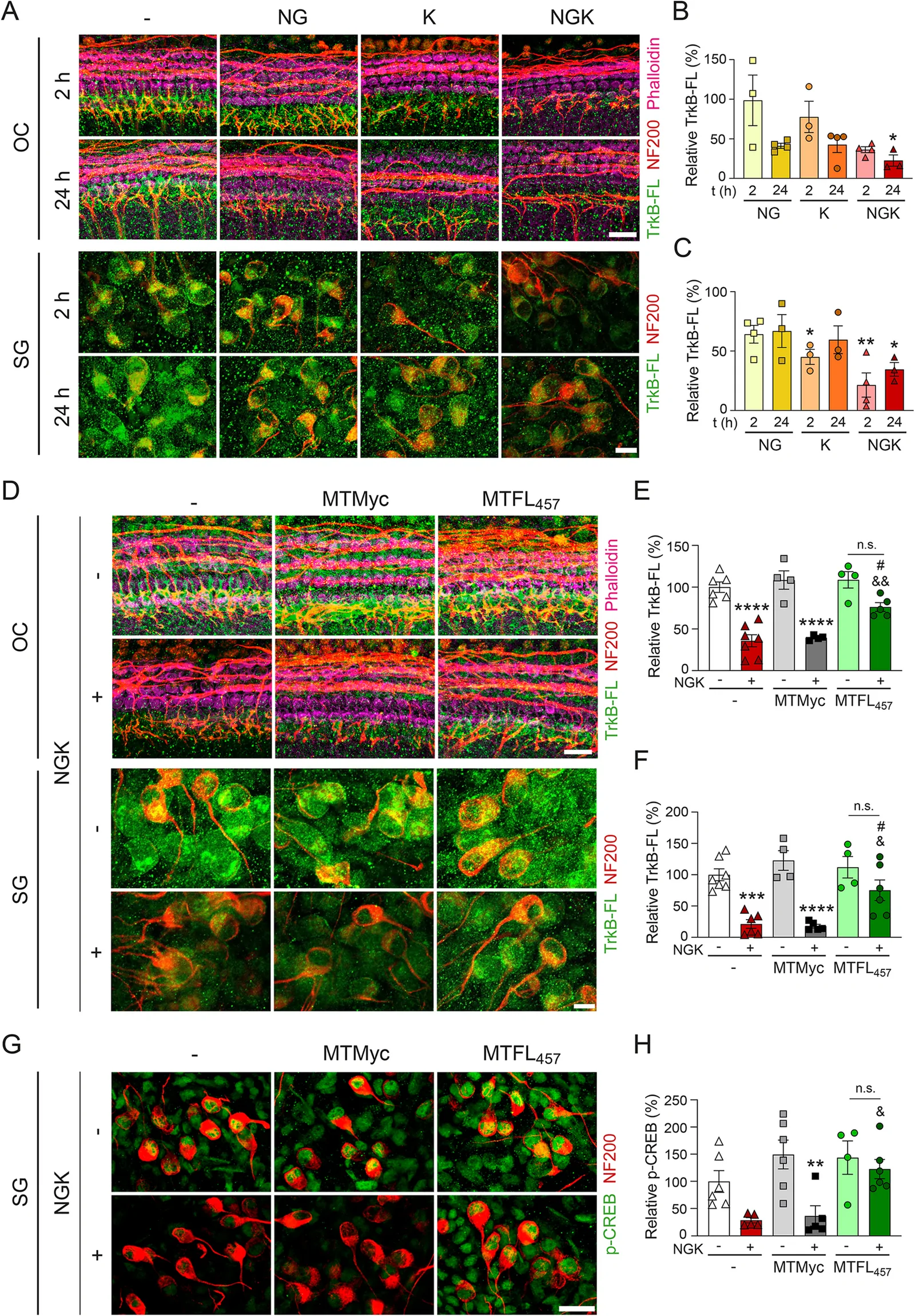
Neuroprotection in ex vivo excitotoxicity after MTFL_457_ pre-treatment derives from stabilization of TrkB-FL receptor and downstream signaling. **A** Representative confocal images showing TrkB-FL receptor levels in the organ of Corti (OC, upper panels) and spiral ganglion (SG, lower panels) under different excitotoxic conditions. Cochlear explants were treated for 2 h with different GluR agonists (NG, K or NGK), and then fixed 2 or 24 h post-excitotoxic insult and processed for immunofluorescence, focusing on the cochlear middle turn (see Fig. 1A). Explants were labeled for TrkB-FL (green), NF200 (red) and phalloidin (HC apical structures marker, purple). Scale bars: 20 μm (upper panels); 10 μm (lower panels). **B-C** Analysis of TrkB-FL levels, relative to their respective controls (untreated explants, assigned an arbitrary value of 100%), in the OC (**B**) and the SG (**C**) following the induction of the excitotoxic insults. Means ± SEM are represented (3–4 explants/condition). Statistically significant differences were detected by Kruskal-Wallis test followed by Dunn’s multiple comparison test (**B**) or one-way or ANOVA test followed by Tukey’s multiple comparison test (**C**): * K/NGK 2/24 h vs. respective controls (* *P* < 0.05; ** *P* < 0.01). **D** Representative confocal images of TrkB-FL receptor levels in the OC (upper panels) and SG (lower panels) in explants pre-treated with MTFL_457_ or MTMyc (6 h), or no peptide, and then treated with NGK (2 h) as above indicated (Fig. 2A). As additional controls, the induction of excitotoxicity was omitted for some explants. After 24 h, explants were fixed and labeled as previously described. Scale bars: 20 μm (upper panel); 10 μm (lower panel). **E-F** Quantification of TrkB-FL levels, relative to untreated explants (assigned an arbitrary value of 100%), in the OC (**E**) and the SG (**F**). Data are presented as means ± SEM (4–7 explants/condition). Statistical analysis was performed by one-way ANOVA test followed by Tukey’s multiple comparison test: * NGK/ MTMyc-NGK vs. respective control, & MTFL_457_-NGK vs. NGK, # MTFL_457_-NGK vs. MTMyc-NGK (n.s., non-significant; &, # *P* < 0.05; && *P* < 0.01; *** *P* < 0.001; **** *P* < 0.0001). **G** Representative confocal images showing levels of CREB active form, p-CREB, in the SG of explants treated as in panel **D**. Explants were labeled with antibodies for p-CREB (green) and NF200 (red). Scale bar: 20 μm. **H** Evaluation of p-CREB levels, relative to untreated explants (assigned an arbitrary value of 100%), in the SG. Means ± SEM are represented (4–6 explants/condition). Statistically significant differences were detected by one-way ANOVA test followed by Tukey’s multiple comparison test: * MTMyc-NGK vs. respective control, & MTFL_457_-NGK vs. NGK (n.s., non-significant; & *P* < 0.05; ** *P* < 0.01)

The possible relevance of MTFL_457_ for in vivo therapy of NIHL drove us to further investigate this peptide mechanism of action in cochlear explants. Previous studies had demonstrated that, in cortical neurons, excitotoxicity promoted TrkB-FL receptor endocytosis and retrograde trafficking to the Golgi apparatus, where cleavage by calpain severely compromised BDNF-mediated neurotrophic support. Moreover, these processes could be prevented by treatment with peptide MTFL_457_, both in cultured neurons undergoing excitotoxicity and mice stroke (Tejeda et al. 2019; Esteban-Ortega et al. 2025). Therefore, we first evaluated the effect of excitotoxic damage induced by the different GluR agonists (NG, K and NGK) in TrkB-FL levels in the cochlear explants (Fig. 5A-C; Supplementary Fig. 2E-G), following the experimental design previously described (Fig. 1A; Supplementary Fig. 2A). We observed a strong decrease in levels of TrkB-FL, detected with an isoform-specific antibody, with all excitotoxic treatments tested, both at the organ of Corti (particularly in SGN synaptic terminals) (Fig. 5A, upper panels, and B) and the spiral ganglion (Fig. 5A, lower panels, and C). However, this effect was more pronounced following NGK treatment which yielded a significant reduction in receptor levels after damage induction, exceeding 50% compared to the untreated explants, both at 2 h (spiral ganglion) and 24 h (organ of Corti and spiral ganglion). A tendency toward reduced TrkB-FL levels in the organ of Corti (Supplementary Fig. 2E, upper panels, and F) and the spiral ganglion (Supplementary Fig. 2E, lower panels, and G) was also observed 48 h after damage induction.

Once demonstrated that TrkB-FL downregulation is also produced in cochlear explants under excitotoxic conditions, we next sought to investigate if MTFL_457_ peptide also stabilized this receptor. Thus, cochlear explants were pre-treated with MTMyc or MTFL_457_, or left untreated, before NGK treatment for induction of excitotoxicity (Fig. 5D-F), following the experimental design previously described (Fig. 2A). MTFL_457_ partially prevented the excitotoxicity-induced decrease in TrkB-FL, maintaining the receptor levels at approximately 75% compared to untreated explants, both at the organ of Corti (particularly in SGN synaptic terminals) (Fig. 5D, upper panels, and E) and the spiral ganglion (Fig. 5D, lower panels, and F). In contrast, this effect did not occur in the explants treated with MTMyc + NGK or NGK alone where, once again, a significant decrease of receptor levels greater than 50% was observed in both regions. Finally, we investigated if TrkB-FL downstream signaling was also recovered in cochlear explants as a consequence of receptor stabilization by MTFL_457_ action. An important TrkB-FL downstream effector in neurotrophin signaling is CREB transcription factor, which is involved in a feedback survival mechanism triggered by MTFL_457_ in cortical neurons (Tejeda et al. 2019). Therefore, we analyzed the levels of Ser 133 phosphorylated CREB (p-CREB), considered as the active form of this prosurvival transcription factor, in explants treated as before (Fig. 2A). Excitotoxicity induced a significant decrease in p-CREB levels exceeding 65% in the spiral ganglion, in the absence of peptide or with MTMyc. In contrast, the levels of active transcription factor were notably preserved in MTFL_457_-pre-treated explants (Fig. 5G-H). Altogether, these results demonstrate that glutamate excitotoxicity, particularly that induced by the combined NGK treatment, disrupts cochlear neurotrophic support by destabilizing the TrkB-FL receptor. Nevertheless, treatment with MTFL_457_ is able to partially preserve the receptor levels and, consequently, maintain prosurvival signaling cascades in the cochlea. These results strongly suggest that stabilizing BDNF/TrkB-FL signaling may represent the primary mechanism underlying MTFL_457_-mediated neuroprotection in the cochlea.

### Peptide MTFL_457_ is neuroprotective in a noise-induced model of hearing loss

**Fig. 6:**
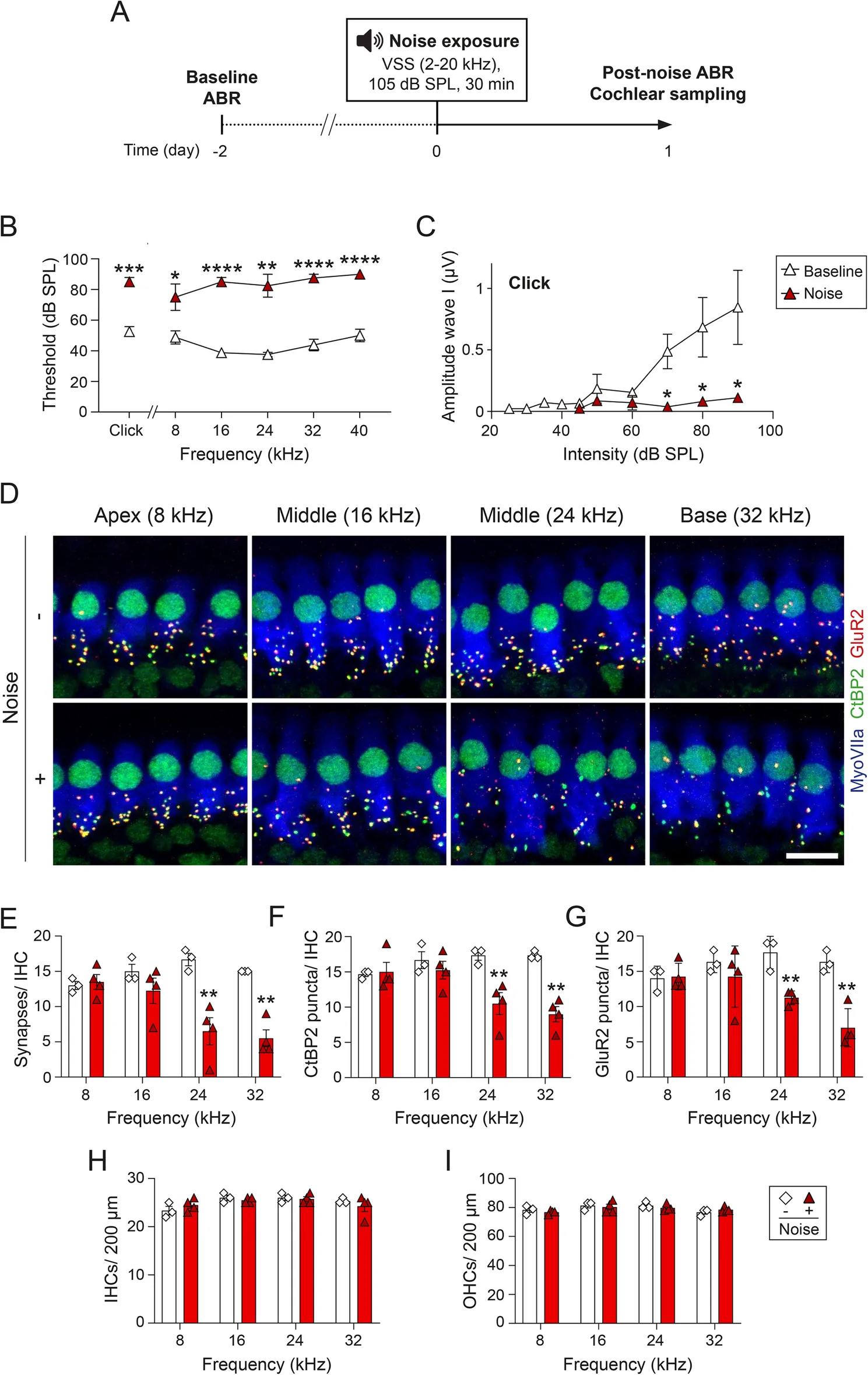
Hearing dysfunction and cochlear synaptopathy are induced in an in vivo model of noise overexposure. **A** Experimental design. Two-month-old C57BL/6j male mice were exposed to violet swept sine (VSS) noise at 105 dB SPL for 30 min. The effect of noise exposure on hearing was evaluated by the ABR test before (baseline) and 1 day after exposure. At this time point, animals were sacrificed and cochleae were collected. Cochleae from control unexposed animals were also sampled. **B** ABR thresholds in response to click and tone-burst stimuli (8, 16, 24, 32 and 40 kHz) at baseline and 1 day after exposure. **C** Evaluation of ABR wave I amplitude plotted against click stimulus intensity (dB SPL). **B-C** Means ± SEM are represented (*n* = 4 animals). Statistically significant differences for each sound stimuli (**B**) or each intensity (**C**) were analyzed by two-tailed Student’s t test: * noise vs. baseline (* *P* < 0.05; ** *P* < 0.01; *** *P* < 0.001; **** *P* < 0.0001). **D** Representative confocal images of cochlear synapses in the organ of Corti at apical (8 kHz), middle (16 and 24 kHz) and basal (32 kHz) turns in noise-exposed and control animals 1 day after exposure. Whole mount preparations were immunolabeled with antibodies for MyoVIIa (blue), CtBP2 (green), and GluR2 (postsynaptic marker, red). Scale bar: 10 μm. **E-F** Quantification of synapses (colocalized CtBP2/GluR2 puncta, **E**) and total CtBP2 (**F**) and GluR2 (**G**) puncta per IHC. Data are presented as means ± SEM (*n* = 3–4 animals/group). Statistical analysis was performed by two-tailed Student’s t test: * noise vs. control unexposed animals (** *P* < 0.01). **H-I** Quantification of IHCs (**H**) and OHCs (**I**) over a 200 μm segment of the basilar membrane at apical (8 kHz), middle (16 and 24 kHz) and basal (32 kHz) turns. Means ± SEM are represented (*n* = 3–4 animals/group). Statistical analysis was established by two-tailed Student’s t test. No statistically significant differences were found between noise-exposed and control animals

Our next objective was to investigate if, similarly to the ex vivo excitotoxicity model, MTFL_457_ might also have a neuroprotective effect in vivo. We decided to use a NIHL model where the excitotoxic damage is induced in mice by overexposure to noise, causing excessive glutamate release and GluRs overactivation, followed by a cascade of deleterious processes impacting cochlear structural integrity and hearing function (Kujawa and Liberman 2009; Varela-Nieto et al. 2020). First, we validated a NIHL model in C57BL/6j male mice which involves an exposure to 105 dB VSS noise for 30 min, as previously described by our group (Sanz et al. 2015). Hearing function was evaluated by the auditory brainstem responses (ABR) test before (baseline) and 1 day after noise overexposure (Fig. 6A). As expected, auditory thresholds were significantly increased following noise exposure, both for click stimuli and tone-bursts. For tone-bursts, the threshold shift was more pronounced at mid (16–24 kHz) and high (32–40 kHz) frequencies compared to low frequencies (8 kHz) (Fig. 6B). To assess sound conduction efficiency, we measured the amplitude of wave I, which reflects the peripheral component of the auditory pathway. Noise exposure also caused a significant reduction in wave I amplitude at 70–90 dB SPL click stimulation (Fig. 6C). Because wave I amplitude correlates with the efficiency of sound transmission in the peripheral auditory pathway (Kujawa and Liberman 2009), this decrease likely indicated impaired synaptic transmission between IHCs and SGNs.

The impact of noise on IHC synapse integrity had not been previously assessed in this NIHL model. To address this, we collected cochleae 1 day after noise exposure and performed whole-mount dissection of the organ of Corti, followed by immunolabeling of presynaptic ribbons with anti-CtBP2 and postsynaptic glutamate receptor patches with anti-GluR2 (Fig. 6D). Colocalization of these markers was analyzed along the tonotopic axis, including apical (8 kHz), middle (16 and 24 kHz), and basal (32 kHz) turns. While the more apical regions (8 and 16 kHz) remained unaffected, noise exposure induced a significant reduction in cochlear synapses, exceeding 60%, in regions corresponding to 24 and 32 kHz (Fig. 6E). Both presynaptic and postsynaptic structures were compromised, as evidenced by a significant decrease in total CtBP2 puncta (Fig. 6F) and GluR2 puncta per IHC (Fig. 6G). In contrast, noise overexposure did not affect hair cell viability, as no significant changes were observed in the number of IHCs (Fig. 6H) or OHCs (Fig. 6I).

**Fig. 7:**
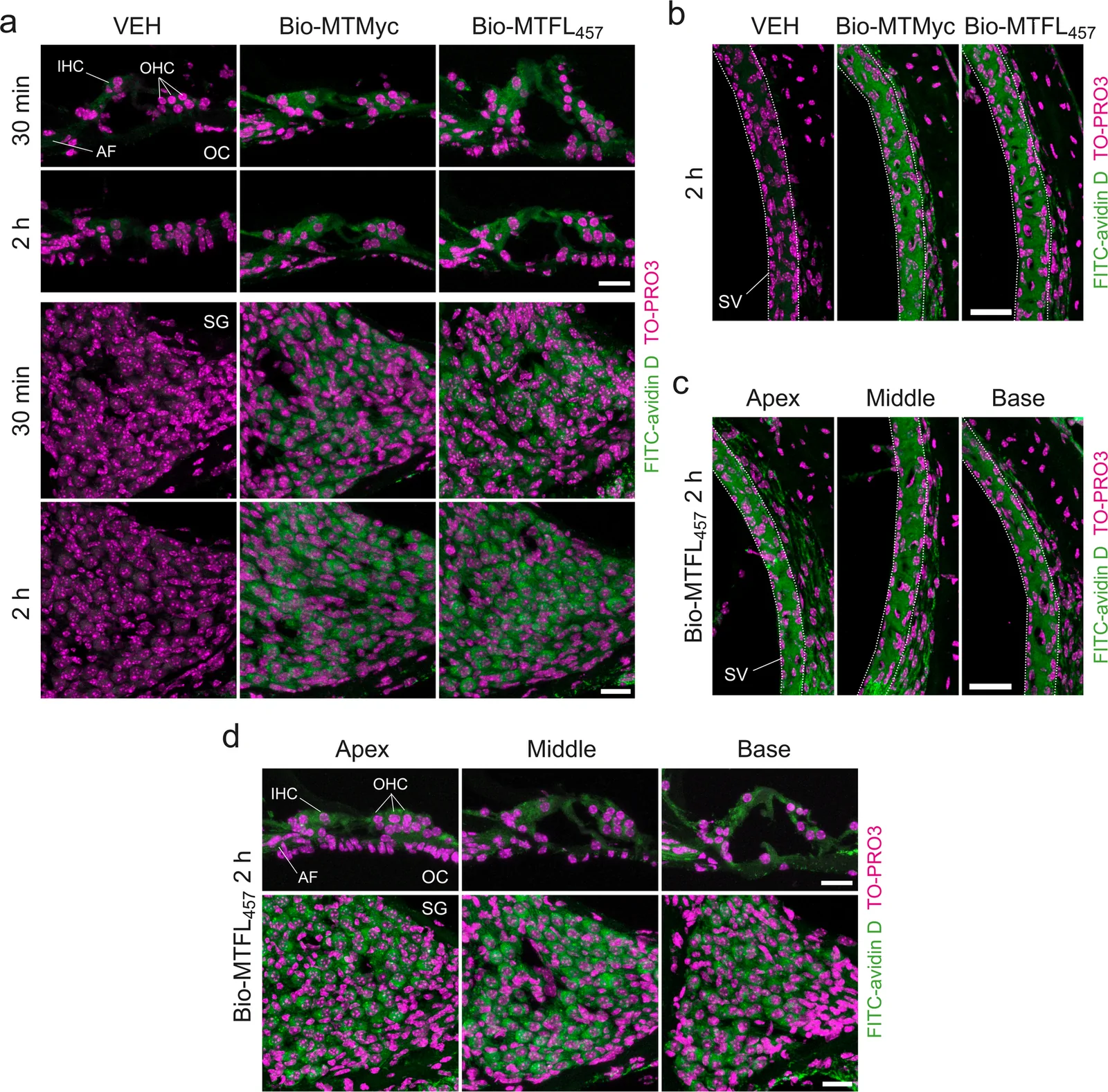
Designed CPPs penetrate and distribute through the inner ear after in vivo administration. **A** Representative confocal images showing Bio-MTFL_457_ and Bio-MTMyc distribution in the Organ of Corti (OC, upper panels) and spiral ganglion (SG, lower panels) within the middle cochlear turn. Mice were i.p. injected with biotinylated peptides (10 nmol/g) or vehicle (saline) and subsequently sacrificed 30 min or 2 h after administration. Cochlear cryosections were stained with FITC-conjugated avidin D (CPP detection, green) and TO-PRO3 (nuclei marker, purple). IHC (inner hair cells), OHC (outer hair cells) and AF (afferent fibers) are indicated. **B** Representative confocal images of Bio-MTFL_457_ and Bio-MTMyc labeling in the stria vascularis (SV, outlined with a dashed line) of the middle turn of the cochlea, 2 h after CPP administration. **C** Representative confocal images of Bio-MTFL_457_ distribution in the SV (outlined as before) across the different cochlear turns, 2 h after administration. **D** Representative confocal images of Bio-MTFL_457_ labeling in the OC (upper panels) and the SG (lower panels) across the different cochlear turns, 2 h after administration. IHC, OHC and AF are indicated as before. Qualitative analysis of Bio-MTFL_457_ and Bio-MTMyc labeling was assessed relative to vehicle animals in 2–4 equivalent cryosections per animal from at least two mice per condition. Bio-MTFL_457_ distribution across the different cochlear turns was similarly evaluated. Scale bars: 20 μm

**Fig. 8:**
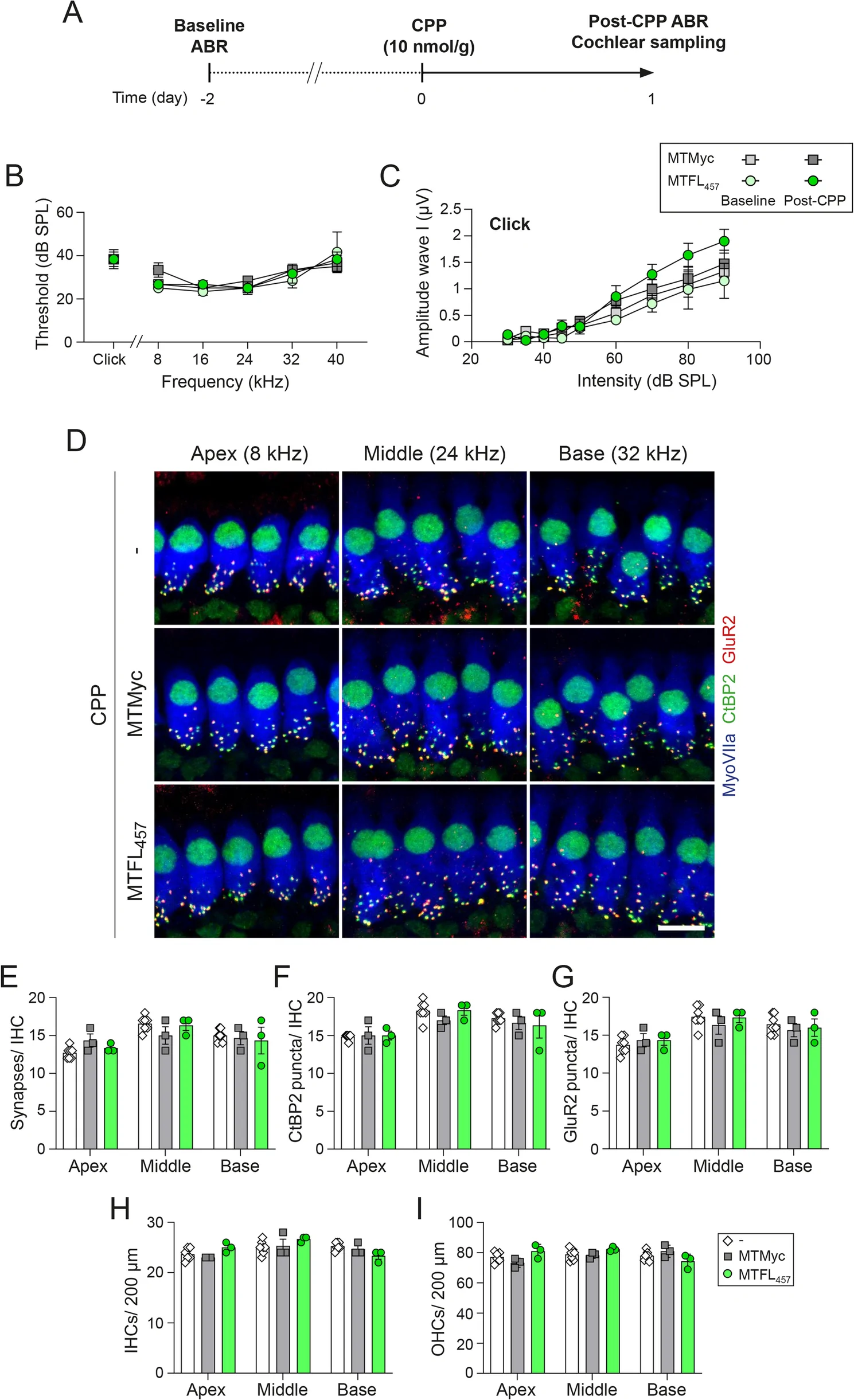
Treatment with CPPs does not affect baseline auditory function or the integrity of cochlear synapses and HCs. **A** Experimental design. Two-month-old C57BL/6j male mice were i.p. injected with MTFL_457_ or MTMyc (10 nmol/g). Auditory function was evaluated by the ABR test before (baseline) and 1 day after treatment. At this time point, animals were sacrificed and cochleae were collected. Cochleae from control untreated animals were also sampled. **B** ABR thresholds in response to click and tone-burst stimuli (8, 16, 24, 32 and 40 kHz) before (baseline) and after CPP treatment. **C** Analysis of ABR wave I amplitude plotted against click stimulus intensity (dB SPL). (**B-C**) Data are presented as means ± SEM (*n* = 3 animals/group). Statistical analysis for each sound stimuli (**B**) or each intensity (**C**) was performed by one-way ANOVA test followed by Tukey’s multiple comparison test. No significant changes in auditory function were observed after CPP treatment. **D** Representative confocal images of cochlear synapses in the organ of Corti at apical (8 kHz), middle (24 kHz) and basal (32 kHz) turns in CPP-treated and control animals 1 day after treatment. Whole mount preparations were labeled with antibodies for MyoVIIa (blue), CtBP2 (green), and GluR2 (red). Scale bar: 10 μm. **E-G** Quantification of synapses (**E**) and total CtBP2 (**F**) and GluR2 (**G**) puncta per IHC. **H-I** Analysis of IHC (**H**) and OHC (**I**) counts over a 200 μm segment of the basilar membrane at apical (8 kHz), middle (24 kHz) and basal (32 kHz) turns. **E-I** Data are presented as means ± SEM (*n* = 3–7 animals/group). Statistical analysis was assessed by one-way ANOVA test followed by Tukey’s multiple comparison test. No significant differences were found between CPP-treated and control animals for any of the parameters analyzed

After validating the NIHL model for studying noise-induced cochlear synaptopathy, and prior to evaluating the potential neuroprotective effect of MTFL_457_, we examined the ability of this CPP to penetrate and distribute throughout the inner ear, as well as its biosafety following systemic administration. Male mice received intraperitoneal (i.p.) injections of biotinylated peptides, Bio-MTMyc or Bio-MTFL_457_ (10 nmol/g), or vehicle (saline), and were sacrificed 30 min or 2 h later. Analysis of cochlear cryosections using FITC-conjugated avidin D revealed specific localization of these peptides in the organ of Corti and spiral ganglion within the middle turn at both time points (Fig. 7A). Detection of Bio-MTMyc and Bio-MTFL_457_ in the stria vascularis 2 h post-injection confirmed their ability to cross the blood-labyrinth barrier and reach the cochlea (Fig. 7B). Furthermore, Bio-MTFL_457_ exhibited a relatively uniform distribution across the stria vascularis (Fig. 7C), organ of Corti, and spiral ganglion (Fig. 7D) in all cochlear turns. We next evaluated potential MTMyc or MTFL_457_ adverse effects in auditory function and cochlear integrity under basal conditions by analyzing male mice i.p. injected with these peptides (10 nmol/g), or no peptide, 1 day after administration (Fig. 8A). Neither peptide induced significant ABR threshold shifts for click or pure-tone stimuli (Fig. 8B), nor did they affect wave I amplitude at any click stimulus intensity (Fig. 8C). Similarly, synapse counts (Fig. 8D-E) and total CtBP2 (Fig. 8F) or GluR2 (Fig. 8G) puncta per IHC remained unchanged across cochlear turns. Finally, the numbers of IHCs (Fig. 8H) and OHCs (Fig. 8I) did not differ significantly among groups, confirming the biosafety of both CPPs under basal conditions.

**Fig. 9:**
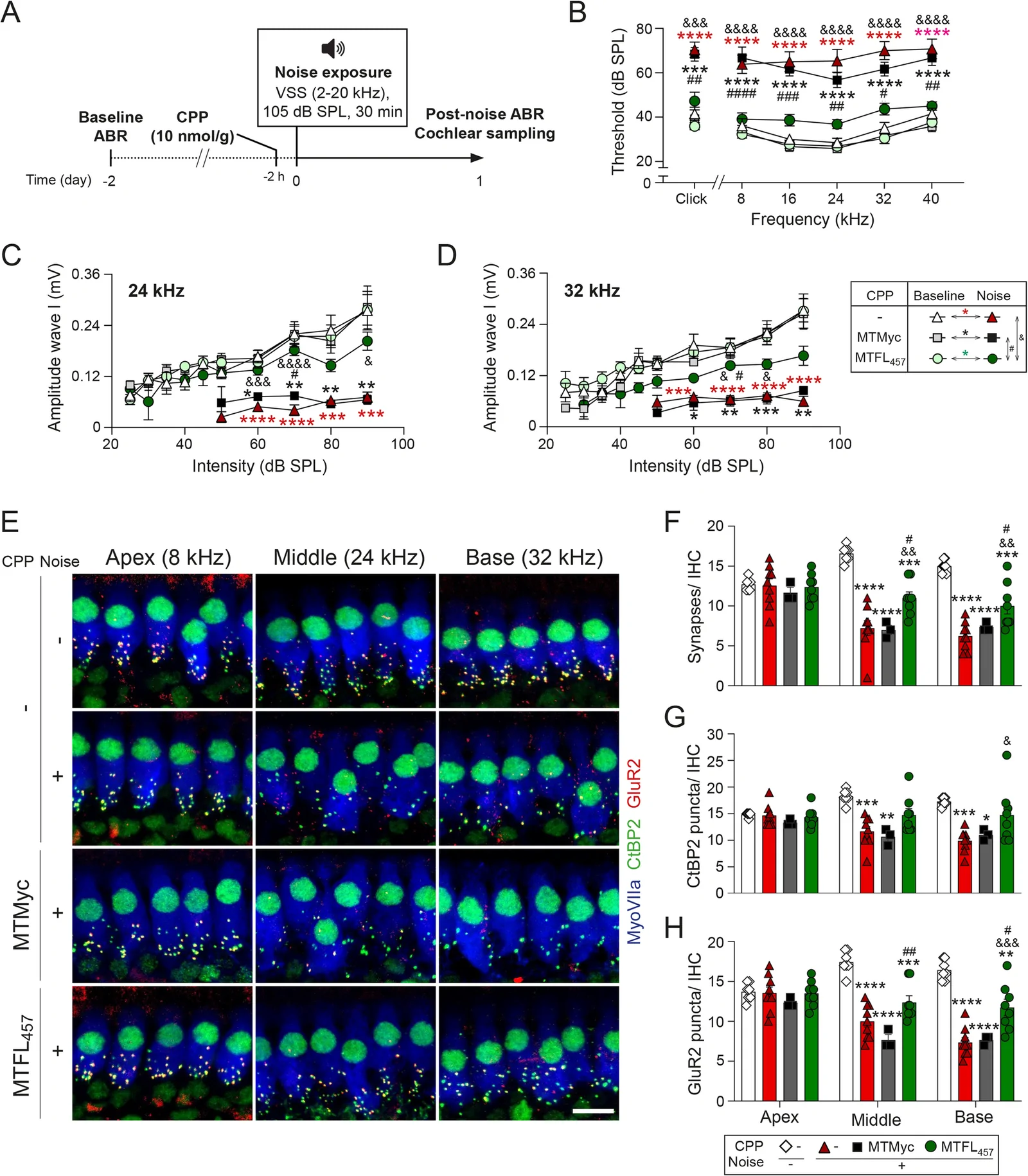
MTFL_457_ pre-treatment prevents NIHL and cochlear synaptopathy in male mice. **A** Experimental design. Two-month-old C57BL/6j male mice were i.p. injected with MTFL_457_ or MTMyc (10 nmol/g), or left untreated, 2 h before exposure to VSS noise at 105 dB SPL for 30 min. Hearing function was evaluated by the ABR test before (baseline) and 1 day after CPP pre-treatment and noise exposure. At this time point, animals were sacrificed and cochleae were collected. Cochleae from control animals (untreated and unexposed to noise) were also sampled. **B** ABR thresholds in response to click and tone-burst stimuli (8, 16, 24, 32 and 40 kHz) before (baseline) and 1 day after exposure in CPP-pre-treated or untreated animals. Means ± SEM are represented (*n* = 6–13 animals/group). **C-D** Analysis of ABR wave I amplitude plotted against stimulus intensity (dB SPL) for 24 kHz (**C**) and 32 kHz (**D**) tone-bursts. Means ± SEM are represented (*n* = 6–10 animals/group). **B-D** Statistical analysis for each sound stimuli (**B**) or each intensity (**C-D**) between the different groups was performed by one-way ANOVA test followed by Tukey’s multiple comparison test: *, noise condition vs. respective baseline in the absence of CPP (red), or in the presence of MTMyc (black) or MTFL_457_ (green); &, MTFL_457_-noise vs. noise; #, MTFL_457_-noise vs. MTMyc-noise (*, #, & *P* < 0.05; **, ## *P* < 0.01; ***, &&&, ### *P* < 0.001; ****, &&&&, #### *P* < 0.0001). **E** Representative confocal images of cochlear synapses in the organ of Corti at apical (8 kHz), middle (24 kHz) and basal (32 kHz) turns in CPP-pre-treated or untreated animals 1 day after noise insult and in control animals (untreated and unexposed). Whole mount preparations were immunolabeled with antibodies for MyoVIIa (blue), CtBP2 (green), and GluR2 (red). Scale bar: 10 μm. **F-H** Quantification of synapses (**F**) and total CtBP2 (**G**) and GluR2 (**H**) puncta per IHC. Data are presented as means ± SEM (*n* = 3–8 animals/group). Statistically significant differences were analyzed by one-way ANOVA test followed by Tukey’s multiple comparison test: * noise/ MTMyc-noise/ MTFL_457_-noise vs. control, & MTFL_457_-noise vs. noise, # MTFL_457_-noise vs. MTMyc-noise (*, &, # *P* < 0.05; **, &&, ## *P* < 0.01; ***, &&& *P* < 0.001; **** *P* < 0.0001)

**Fig. 10:**
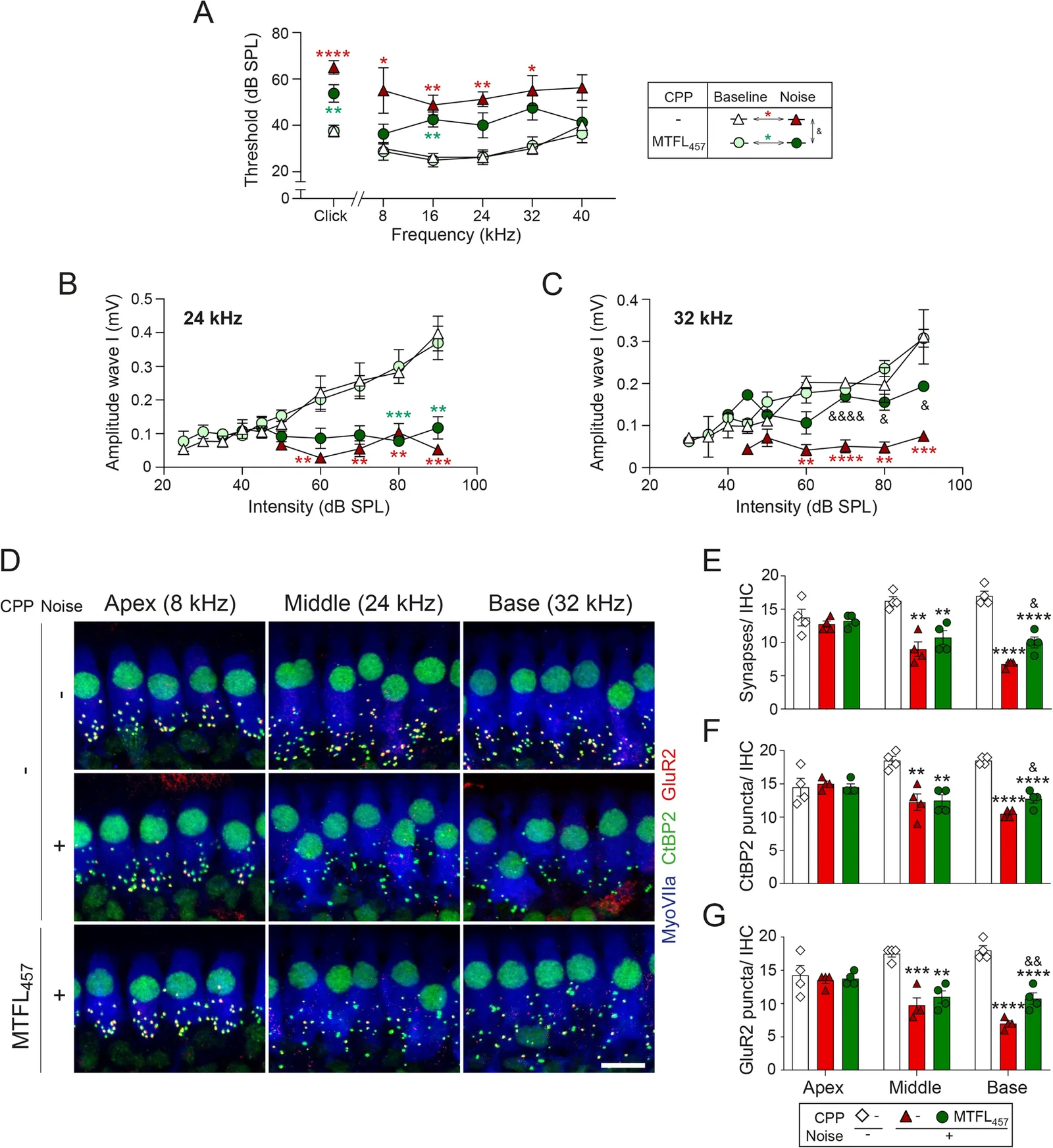
MTFL_457_ pre-treatment prevents NIHL and cochlear synaptopathy in female mice. The experimental design was the same as that described in Fig. 8A but performed in female mice injected with MTFL_457_ or no peptide. **A** ABR thresholds in response to click and tone-burst stimuli (8, 16, 24, 32 and 40 kHz) before (baseline) and 1 day after noise insult in CPP-pre-treated or untreated animals. **B-C** Analysis of ABR wave I amplitude plotted against stimulus intensity (dB SPL) for 24 kHz (**B**) and 32 kHz (**C**) tone-bursts. **A-C** Data are presented as means ± SEM (*n* = 4 animals/group). Statistical analysis for each sound stimuli (**A**) or each intensity (**B-C**) between the different groups was evaluated by one-way ANOVA test followed by Tukey’s multiple comparison test: *, noise condition vs. respective baseline in the absence of CPP (red) or in the presence of MTFL_457_ (green); &, MTFL_457_-noise vs. noise (*, & *P* < 0.05; ** *P* < 0.01; *** *P* < 0.001; ****, &&&& *P* < 0.0001). **D** Representative confocal images of cochlear synapses in the organ of Corti at apical (8 kHz), middle (24 kHz) and basal (32 kHz) turns in MTFL_457_-pre-treated or untreated animals 1 day after exposure and in control animals (untreated and unexposed). Whole mount preparations were immunolabeled with antibodies for MyoVIIa (blue), CtBP2 (green), and GluR2 (red). Scale bar: 10 μm. **E-G** Analysis of the number of synapses (**E**) and total CtBP2 (**F**) and GluR2 (**G**) puncta per IHC. Data are presented as means ± SEM (*n* = 4 animals/group). Statistically significant differences were analyzed by one-way ANOVA test followed by Tukey’s multiple comparison test: * noise/MTFL_457_-noise vs. control, & MTFL_457_-noise vs. noise (& *P* < 0.05; **, && *P* < 0.01; *** *P* < 0.001; **** *P* < 0.0001)

Finally, we evaluated MTFL_457_ neuroprotective potential in the NIHL model. To this aim, male and female mice were i.p. injected with MTFL_457_ (and also MTMyc, in the case of male mice) (10 nmol/g), or left untreated, 2 h before noise overexposure. Hearing was evaluated with the ABR test before (baseline) and 1 day after peptide pre-treatment and noise exposure, the cochleae being collected after that (Fig. 9A). Male mice pre-treated with MTMyc exhibited a significant increase of auditory thresholds after noise exposure (~ 30–35 dB SPL shift), both for the click and the tone-burst stimuli, which was comparable to that of the untreated animals. Conversely, the ABR threshold increase was significantly prevented in MTFL_457_-pre-treated mice (~ 10–15 dB SPL shift) compared to data from MTMyc-pre-treated or untreated animals (Fig. 9B). In MTMyc-pre-treated mice, similarly to untreated animals, the noise insult also induced a significant decrease in the amplitude of wave I at 70–90 dB SPL click stimulation (Supplementary Fig. 4A). In male mice pre-treated with MTFL_457_, although a modest tendency toward higher wave I amplitudes at 80 and 90 dB SPL was observed, these values significantly differed from the baseline recordings of these animals (Supplementary Fig. 4A). Since the click is a broadband stimulus, the specific amplitude of wave I in response to 24 and 32 kHz tone-burst stimuli was also analyzed (Fig. 9C-D). This approach had in consideration our previous observation of a significant synaptic loss induced by noise exposure in cochlear regions corresponding to these frequencies (Fig. 6D-E). Of note, the amplitude of wave I was significantly decreased at 60–90 dB SPL for both 24 kHz (Fig. 9C) and 32 kHz (Fig. 9D) tone-burst stimuli in MTMyc-pre-treated and untreated animals subjected to noise. In contrast, this reduction was prevented by pre-treatment with MTFL_457_, as no significant changes were observed compared to the baseline values (Fig. 9C-D). Accordingly, after noise exposure, the number of cochlear synapses per IHC in MTFL_457_-pre-treated mice, both in the middle (24 kHz) and basal turns (32 kHz), was significantly higher than in MTMyc-pre-treated or untreated animals (Fig. 9E-F). Indeed, MTFL_457_ pre-treatment partially prevented the destabilization of both the presynaptic and postsynaptic regions in the middle and basal turns, evidenced by a partial preservation of total CtBP2 (Fig. 9G) and GluR2 (Fig. 9H) puncta per IHC. Again, MTFL_457_ effect was specific and not observed in animals treated with the control peptide or those untreated. In the case of the female mice, although the increase in auditory thresholds induced by noise exposure showed a tendency toward lower values in MTFL_457_-pre-treated mice (5–15 dB SPL shift) compared to the untreated animals (~ 20–25 dB SPL shift), these differences were no significant (Fig. 10A). This might be partially attributed to the fact that, as previously reported (Lin et al. 2022; Zakaria et al. 2019), the noise-induced ABR threshold shift in female mice was less pronounced than in male mice while baseline values did not differ (Table 1). Although a sexual dimorphism in peptide efficacy cannot be completely excluded, it should be noted that the auditory thresholds after the noise insult were quite similar in male and female mice pre-treated with MTFL_457_ (Table 1), suggesting that the range of hearing function improvement induced by MTFL_457_ might have been more limited for female mice. Upon click stimulation, a similar decrease in the amplitude of wave I at 70–90 dB SPL was noticed in MTFL_457_-pre-treated and untreated animals after noise exposure (Supplementary Fig. 4B), analogous results being obtained in response to a 24 kHz tone-burst stimulus (Fig. 10B). In contrast, the amplitude of wave I at 70–90 dB SPL in response to 32 kHz tone-burst stimulation was significantly higher in female mice pre-treated with MTFL_457_ (Fig. 10C). Accordingly, after noise exposure, the number of synapses per IHC in the basal turn (32 kHz) was significantly higher compared to that of the untreated animals (Fig. 10D-E), demonstrating a protective effect of MTFL_457_ pre-treatment on cochlear synapses of female mice. A similar result was noted in the total number of CtBP2 (Fig. 10F) and GluR2 (Fig. 10G) puncta per IHC which suggests the stabilization of, respectively, presynaptic and postsynaptic structures in the basal turn. 

**Table 1:** Comparative sex-based analysis of ABR auditory thresholds. ABR thresholds were assessed in male and female mice pre-treated or not with MTFL_457_ (10 nmol/g) in response to both click stimuli and tone-burst stimuli (8, 16, 24, 32 and 40 kHz), before (baseline) and 1 day after noise exposure. Data are shown as mean ± SEM (males: n = 6–13; females: n = 4 animals/group). Statistical differences between sexes for each auditory stimulus within the same experimental condition were analyzed using two-tailed Student’s t-test. Although no significant differences were found, a tendency toward a higher increase in auditory thresholds was observed in untreated male mice compared to female mice after noise exposure. However, the ABR thresholds were quite similar in animals of both sexes pre-treated with MTFL_457_ after the noise insult. M, males; F, females

Threshold (dB SPL)								
Stimuli	Noise				MTFL _457_ + noise			
	Baseline		Post-noise		Baseline		Post-noise	
	M	F	M	F	M	F	M	F
Click	41±3	38 ±3	70 ± 5	65 ±3	36 ± 2	38 ± 1	47 ± 4	54 ± 4
8 kHz	36 ±3	30 ± 0	64 ± 4	55 ±10	32 ± 2	29 ± 4	39 ±3	36 ±4
16 kHz	30 ± 2	26 ± 1	65 ± 4	49 ± 4	28 ±2	25 ±3	39 ± 3	42±3
24 kHz	28 ± 2	26 ±3	65 ±5	51 ±3	27 ± 1	26 ±3	37 ± 2	40 ±5
32 kHz	35 ±3	30±2	70 ± 4	55 ± 6	30 ±2	31 ± 4	44 ±3	47 ±5
40 kHz	41 ±2	40± 0	71±2	56±5	38 ± 2	36 ±4	45 ±2	41 ±7

In conclusion, these findings demonstrate that although both MTFL_457_ and its control peptide MTMyc can cross the blood-labyrinth barrier and distribute uniformly throughout the cochlea within 2 h of administration, only MTFL_457_ exhibits neuroprotective effects, confirming that these are specific to the TrkB-FL-derived sequence. In male mice exposed to noise, MTFL_457_ attenuates both HL and cochlear synaptopathy, whereas its efficacy on auditory function in females remains uncertain, despite a consistent protective effect on cochlear synapses. These properties, combined with the absence of adverse effects under basal conditions, strongly support the therapeutic potential of MTFL_457_ for the treatment of NIHL and other forms of HL associated with excitotoxicity and reduced neurotrophic support.

## Discussion

### Excitotoxic cochlear synaptopathy: postsynaptic vulnerability and neurotrophic collapse

Physiological synaptic transmission in the cochlea is glutamatergic, the IHC-released neurotransmitter activating various GluRs on the SGN surface (Niedzielski and Wenthold 1995; Klotz et al. 2019; Caprara and Peng 2022; Coate et al. 2019; Glowatzki et al. 2008). In contrast, noise overexposure triggers excessive glutamate release and leads to GluR overactivation and cochlear excitotoxicity (Kujawa and Liberman 2009), causing synaptic loss and afferent fiber degeneration. Notably, HCs remain largely unaffected unless damage is severe or prolonged (Kujawa and Liberman 2009; Neves et al. 2023; Varela-Nieto et al. 2020). Here, we investigate the mechanisms of cochlear excitotoxicity using an ex vivo model of explants exposed to glutamate analogs (NMDA/glycine, kainate, or their combination). While previous studies emphasized the role of AMPARs and KARs (Juiz et al. 1989; Puel et al. 1991, 1994; Pujol et al. 1985), the contribution of NMDARs is controversial. We demonstrate here that NMDAR overactivation alone induces significant damage and exacerbates AMPAR/KAR-mediated injury, supporting a joint involvement of all three GluR types in cochlear damage. Because NMDAR activation requires co-agonists binding (NMDA and glycine/D-serine) and membrane depolarization (Pagano et al. 2021), previous discrepancies (Puel et al. 1994; Wang and Green 2011; Saidia et al. 2024) likely reflect incomplete NMDAR overactivation in earlier models.

Considering these results, we selected combined GluR activation in explants to accurately mirror in vivo excitotoxicity. This treatment preserves HC viability but induces synaptic loss, primarily through postsynaptic destabilization (reduced PSD‑95 puncta) with largely intact presynaptic ribbons (CtBP2 puncta). Since PSD-95 is a scaffold for large multiprotein complexes, including GluRs (Christopherson et al. 1999) and TrkB-FL receptors (Cao et al. 2013), its dramatic depletion likely disrupts synaptic transmission and prosurvival signaling, having a major impact on SGN structure, survival and function. Indeed, we observe that synaptic terminal destabilization precedes the structural disruption of type I SGN afferent fibers. Furthermore, SGN somata displays time-dependent FJC labeling, consistent with ongoing neuronal degeneration, and Golgi apparatus fragmentation —an organelle hallmark of excitotoxic stress observed across neurodegenerative contexts (Gonatas et al. 2006; Martinez-Menarguez et al. 2019), cerebral ischemia (Tejeda et al. 2019; Esteban-Ortega et al. 2025), and epilepsy (Skupien-Jaroszek et al. 2023). In cortical neurons undergoing excitotoxicity, we have shown that overactivation of NMDARs triggers TrkB‑FL endocytosis, retrograde transport to the Golgi, and calpain‑mediated cleavage, ultimately impairing BDNF signaling and the activity of prosurvival transcription factors, CREB and MEF2 (Tejeda et al. 2019; Esteban-Ortega et al. 2025). However, the mechanistic link between TrkB-FL regulation, calpain activity, and Golgi fragmentation has not yet been directly demonstrated in the inner ear. From previous studies, we infer that TrkB-FL downregulation induced by excitotoxicity in the cochlea similarly promotes dysfunction of the Golgi complex in SGNs, compromising function and viability of these neurons. Moreover, neurotrophic support is also essential for SGNs neurite maintenance and synapse stability (Frago et al. 2000; Green et al. 2012; Ma et al. 2019). Therefore, TrkB‑FL destabilization might be considered as a proximal driver of noise injury, occurring upstream of postsynaptic disassembly, fibers retraction and somata degeneration. These secondary processes would further compromise neurotrophic signaling by hindering access of HC-released neurotrophins to their receptors.

### MTFL_457_ stabilizes TrkB‑FL signaling and interrupts the excitotoxic cascade

MTFL_457_ was designed to disrupt the excitotoxic itinerary of TrkB‑FL to the Golgi apparatus, thereby preventing calpain processing, stabilizing receptor levels on the cell membrane and preserving BDNF prosurvival signaling (Tejeda et al. 2019; Esteban-Ortega et al. 2025). In cochlear explants, MTFL_457_ pre-treatment preserves TrkB‑FL in sensory and neuronal compartments and maintains p‑CREB, indicating sustained prosurvival transcription. Since CREB regulates both BDNF (Tao et al. 1998) and TrkB-FL (Deogracias et al. 2004) expression, MTFL_457_ likely establishes a positive feedback loop that preserves neurotrophic support, maintains Golgi integrity, and reduces neurodegeneration under excitotoxic conditions. Structurally, MTFL_457_ protects SGN afferent integrity and stabilizes postsynaptic architecture by restoring PSD-95 levels without affecting presynaptic ribbons, confirming its postsynaptic selectivity. Mechanistically, TrkB-FL preservation might support the assembly of PSD-95 multiprotein complexes and PSD stabilization by facilitating PSD-95 vesicular transport from the soma to synaptic terminals, a process regulated by BDNF/TrkB signaling (Yoshii and Constantine-Paton 2014). Reciprocally, PSD-95 stabilization might, in turn, promote BDNF-mediated neurotrophic support, as PSD-95/TrkB interactions are essential for optimal receptor function (Cao et al. 2013). Notably, MTFL_457_ also protects cochlear synapses when administered after the excitotoxic insult, identifying a clinically relevant window for therapeutic synaptic repair.

### Translation to in vivo NIHL: overcoming delivery barriers and addressing sex‑specific responses

Therapeutic access to the inner ear is limited by cochlear encapsulation, membrane impermeability and the blood-labyrinth barrier (Nyberg et al. 2019). Here, we demonstrate that biotinylated MTFL_457_ overcomes these limitations. This peptide, and the control peptide MTMyc, reach the stria vascularis, organ of Corti, and spiral ganglion within 2 h of i.p. administration, confirming blood-labyrinth barrier crossing and uniform cochlear distribution. Neither peptide alters ABR thresholds, wave I amplitudes, synapse counts, or HC numbers under basal conditions, supporting the use of MTFL_457_ for cochlear therapy. We assessed MTFL_457_ therapeutic potential in a previously established NIHL model (Sanz et al. 2015) where in vivo excitotoxicity is a key pathogenic mechanism. Although we focus on early outcomes (1 day post-exposure), prior work showing auditory recovery within two weeks supports this paradigm as a temporary NIHL model. While baseline auditory thresholds are comparable between sexes, females show a trend toward lower threshold shifts following noise exposure, consistent with previously described sex-dimorphism in noise vulnerability (Milon et al. 2018; Shuster et al. 2019). Importantly, decreased click-evoked wave I amplitudes in both sexes suggest impaired synaptic transmission at the level of the peripheral auditory pathway. Indeed, this functional deficit is paralleled by a pronounced synaptic loss in the middle (24 kHz) and basal (32 kHz) cochlear turns (Shuster et al. 2019), without compromising HC viability. Noise exposure destabilizes the postsynaptic compartment, consistent with the ex vivo results, although ribbon synapses are also affected in vivo, probably reflecting the fact that HCs are primary targets of noise exposure.

Pre-treatment with MTFL_457_ efficiently prevents threshold shifts in males 1 day post-exposure, whereas early functional recovery in females is less evident, implying a possibly narrower improvement range at early time points. Notably, post‑insult thresholds in MTFL_457_‑treated animals of both sexes converge, suggesting that the peptide’s protective potential might not be sex‑restricted but interact with sex‑dependent damage kinetics. While MTFL_457_ has limited effect on click-evoked wave I amplitudes, it significantly enhances responses to pure tones, particularly at 24 and 32 kHz in males and 32 kHz in females. This frequency-specific improvement likely reflects partial preserved synaptic function in tonotopically restricted regions of the cochlea, more readily detected with narrowband stimuli than with broadband clicks. Consistently, MTFL_457_ confers early and pronounced synaptic protection in both the middle and basal turn in males, and the basal turn in females, stabilizing both pre- and postsynaptic compartments. Nonetheless, in the present study, the interpretation of sex-specific responses is substantially limited by the relatively small size of the female cohort and the absence of a control group treated with peptide MTMyc. Therefore, studies including larger and adequately powered female cohorts, as well as MTMyc-treated animals, will be required to: (1) exclude potential effects of the Tat sequence on female auditory biology; (2) robustly establish whether a true sex-dimorphism in noise vulnerability exists in this NIHL model, which could result in a reduced therapeutic window for MTFL_457_ and (3) further elucidate sex-dependent dynamics and underlying mechanisms (e.g., hormonal milieu, inflammatory and oxidative responses, or GluR expression). Taken together, these findings indicate that MTFL_457_ exerts rapid and robust otoprotective effects in NIHL by preserving hearing function and cochlear synapses, supporting its potential as a therapeutic strategy. However, additional important issues remain to be addressed prior to clinical translation. In particular, future studies should incorporate longitudinal analysis to assess the durability of synaptic protection and functional recovery beyond 1 day after noise exposure. From a mechanistic perspective, such studies will be essential to determine whether MTFL_457_ delays the onset of damage, reduces its magnitude or, alternatively, enhances recovery processes. In addition, pharmacokinetic profiling of this peptide (including tissue distribution, half‑life and clearance) will be required to optimize dosing regimens and refine dose–response relationships. Finally, deeper pathway mapping (e.g., MEF2 activation, transcriptomics and GA integrity biomarkers) and in vivo analysis of post-treatment and post‑insult windows will strengthen causal inference and, by defining realistic intervention timelines, guide clinical translation.

### Positioning MTFL_457_ among neurotrophin‑targeted interventions

Prior strategies to repair cochlear synapses included exogenous BDNF/NT‑3 (Sly et al. 2016; Wan et al. 2014), small‑molecule Trk agonists (Yu et al. 2013; Kempfle et al. 2021) and monoclonal antibodies (Szobota et al. 2019; Vink et al. 2023). Clinical translation has been limited by delivery constraints and the likelihood of TrkB‑FL downregulation after excitotoxic damage, thus reducing the substrate for ligand action. Peptide MTFL_457_ addresses this bottleneck by stabilizing the receptor itself, preserving signaling competence under injury conditions and achieving systemic access across the blood-labyrinth barrier. Its post‑insult efficacy further supports real‑world feasibility where prophylactic intervention is impractical. Mechanistically, MTFL_457_ could be combined with alternative therapies, e.g., selective blockade of Ca²⁺‑permeable AMPARs, antioxidants or anti‑inflammatory agents, to target parallel injury axes (excitotoxic, oxidative, inflammatory) (Varela-Nieto et al. 2020) and potentially yield additive or synergistic protection.

Ultimately, this work supports peptide MTFL_457_ as a compelling candidate for therapy of NIHL and, potentially, other HL forms including ARHL which share excitotoxic and neurotrophic failure mechanisms. If successful, MTFL_457_ could help to close a long‑standing therapeutic gap by targeting a proximal node, TrkB‑FL integrity, that integrates excitotoxic injury and neurotrophic support at the vulnerable cochlear synapse.

## Conclusions

This study identifies MTFL_457_, a TrkB‑FL-derived peptide, as a mechanism‑guided neuroprotectant that preserves cochlear synapses and auditory function in complementary ex vivo and in vivo models of glutamatergic excitotoxicity and NIHL. By stabilizing the BDNF receptor TrkB-FL, this peptide sustains downstream prosurvival signaling and mitigates hallmark features of neurodegeneration. Notably, MTFL_457_ crosses the blood-labyrinth barrier after systemic delivery and has a favorable biosafety profile. Altogether, these findings highlight MTFL_457_ as a promising therapy for NIHL and potentially other sensorineural HL etiologies driven by excitotoxic injury and impaired neurotrophic support.

## Supplementary Information

Supplementary Material 1: Supplementary Table-S1. Key Resources Table.

Supplementary Material 2: Supplementary Fig. 1 *Ex vivo *cochlear explants maintain the characteristic cellular architecture. A Representative confocal image of a whole cochlear explant showing the three cochlear regions: apex, middle, and base. Cochlear explants incubated for 48 h were fixed and labeled with antibodies for MyoVIIa (blue) and NF200 (red) to visualize, respectively, hair cells (HCs) and spiral ganglion neurons (SGNs). B-C Details of a cochlear explant at higher magnification illustrating the preservation of the characteristic cellular organization of the cochlea. B HCs innervated by afferent fibers (AF), originating from SGNs, can be clearly distinguished. C The spatial organization of the outer hair cells (OHCs) and inner hair cells (IHCs) is shown in detail, with OHCs organized in three rows and IHCs in a single row, the latter contacted by the synaptic terminals (ST) of SGNs. Scale bars: (A) 50 μm; (B) 20 μm; (C) 10 μm. Supplementary Fig. 2 Excitotoxicity-induced neurodegeneration and TrkB-FL destabilization 48 h after damage induction. A Experimental design. Cochlear explants were treated for 2 h with different GluR agonists: NG, K or NGK. Subsequently, explants were fixed 48 h after excitotoxicity induction and processed for immunofluorescence, focusing on the cochlear middle turn. B Representative confocal images showing the excitotoxicity-induced degeneration of synaptic terminals and afferent fibers of SGNs, labeled for NF200 (red). Asterisk and arrowheads in NGK-treated explants indicate, respectively, the absence or presence of fibers and synaptic terminals. Qualitative analysis of the structural integrity of cochlear afferent innervation was performed using 3-4 explants per experimental condition. Scale bar: 20 μm. C Representative confocal images showing spiral ganglion neurodegeneration induced by different excitotoxic insults in explants stained with FJC (green) and labeled for NF200 (red). D Analysis of FJC marker levels, relative to the controls (untreated explants, assigned an arbitrary value of 1). Data are presented as mean ± SEM (n = 4 explants/condition). Statistically significant differences were established using Kruskal-Wallis test followed by Dunn’s multiple comparison test: NGK vs control (* P < 0.05). E Representative confocal images showing the effect of different excitotoxic treatments on TrkB-FL receptor levels in the organ of Corti (OC, upper panel) and spiral ganglion (SG, lower panel). Explants were labeled for TrkB-FL (green), NF200 (red) and phalloidin (purple). Scale bars: 20 μm (upper panel); 10 μm (lower panel). F-G Evaluation of TrkB-FL levels, relative to the controls (untreated explants, assigned an arbitrary value of 100%), in the OC (F) and the SG (G) following the induction of the excitotoxic insults. Means ± SEM are represented (3-4 explants/condition). Statistical analysis was performed by Kruskal-Wallis test followed by Dunn’s multiple comparison test (F) or one-way ANOVA test followed by Tukey’s multiple comparison test (G). Although no significant differences were found, a tendency toward a decrease in TrkB-FL levels was observed in all the treatments. Supplementary Fig. 3 Designed CPPs penetrate and distribute through cochlear explants. A Sequence of peptides MTFL_457_, MTMyc, Bio-MTFL_457_, and Bio-MTMyc. The different peptides contain a short sequence belonging to protein Tat (aa 47–57; gray, italic) from HIV-1, followed by the indicated rat TrkB-FL (green, bold) or c-Myc (black, bold) sequences. The four CPPs contain an amido (-NH2) at their C-ter. At the N-ter, MTFL_457_ and MTMyc are modified by an acetyl (Ac-) group while their biotinylated forms, respectively Bio-MTFL_457_ and Bio-MTMyc, contain a biotin moiety (Biotin–Ahx). B-C Representative confocal images of Bio-MTFL_457_ and Bio-MTMyc biodistribution in the organ of Corti (B) and the spiral ganglion (C) within the middle cochlear turn. Explants were incubated with the biotinylated peptides (25 μM) for 2, 6 or 24 h and subsequently fixed and stained with fluorescein (FITC)-conjugated avidin D (green) for CPP detection. A qualitative analysis of Bio-MTFL_457_ and Bio-MTMyc signal was performed from at least three explants per experimental condition. Scale bars: 20 μm. Supplementary Fig. 4 Effect of MTFL_457_ on click-evoked wave I amplitude following noise exposure. Analysis of wave I amplitude plotted against click stimulus intensity (dB SPL) in male (A) and female (B) mice pre-treated or not with MTMyc (in the case of males) or MTFL_457_ (10 nmol/g) before (baseline) and 1 day after noise exposure. Means ± SEM are represented (males, n = 6-11 animals/group; females, n = 4 animals/group). Statistical analysis for each intensity between the different groups was performed by one-way ANOVA test followed by Tukey’s multiple comparison test: *, noise condition vs respective baseline in the absence of CPP (red), or in the presence of MTMyc (black) or MTFL_457_ (green) (*, P < 0.05; **, P < 0.01; ***, P < 0.001; ****, P < 0.0001).

## Data Availability

The data underlying Figs. 1, 2, 3, 4, 5, 6, 8, 9 and 10, and Supplementary Figs. 2 and 4 have been deposited in a public repository (Digital CSIC, http://hdl.handle.net/10261/424340, http://doi.org/10.20350/DIGITALCSIC/18196) and will be made openly accessible prior to publication.

## Abbreviations

ABR: Auditory brainstem responses
AMPARs: Alpha-amino-3-hydroxy-5-methyl-4-isoxazole propionate receptors
ARHL: Age-related hearing loss
BDNF: Brain-derived neurotrophic factor, CPP, cell-penetrating peptide
CREB: CAMP response-element binding protein
FJC: Fluoro-Jade C
GluRs: Glutamate receptors
HCs: Hair cells
HHL: Hidden hearing loss
HL: Hearing loss
IHCs: Inner hair cells
K: Kainate
MEF2: Myocyte enhancer factor 2
KARs: Kainate receptors
NF200: Neurofilament 200 kDa
NT-3: Neurotrophic factor 3
NG: NMDA and glycine
NGK: NMDA, glycine and kainate
NMDARs: N-methyl-D-aspartate receptors
NIHL: Noise-induced hearing loss
OHCs: Outer hair cells
PSD: Postsynaptic density
SGNs: Spiral ganglion neurons
TrkB-FL: TrkB full-length
VSS: Violet swept-sine noise
