# Supplementary material for: Neuroprotective potential of a TrkB-FL-derived cell-penetrating peptide in cochlear synaptopathy and noise-induced hearing loss
Source: Mol Med. 2026 Jun 29;32:148. doi: 10.1186/s10020-026-01539-9 (PMC13584400; doi:10.1186/s10020-026-01539-9)
Supplement: Supplementary file 1 [file 10020_2026_1539_MOESM1_ESM.pdf]

Supplementary Material 1

Neuroprotective potential of a TrkB-FL-derived cell-penetrating peptide in cochlear synaptopathy and noise-induced hearing loss

Authors:

Elena Torres-Campos[a], Isabel Varela-Nieto[a,b,c,*] and Margarita Díaz-Guerra[a,*]

Affiliations:

[a]Instituto de Investigaciones Biomédicas Sols-Morreale (IIBM), Consejo Superior de Investigaciones Científicas-Universidad Autónoma de Madrid, Madrid 28029, Spain

[b]Centro de Investigación Biomédica en Red en Enfermedades Raras (CIBERER), Instituto de Salud Carlos III (ISCIII), Madrid 28029, Spain

[c]Instituto de Investigación Sanitaria del Hospital La Paz (IdiPAZ), Madrid 28046, Spain.

*Corresponding authors: mdiazguerra@iib.uam.es (ORCID ID: 0000-0002-4478-0956) and i.varela.nieto@csic.es (ORCID ID: 0000-0003-3077-0500)

Supplementary Table S-1. Key Resources Table. Description of reagents or resources used in the study with source and identifier

REAGENT or RESOURCE	SOURCE	IDENTIFIER
Antibodies		
Rabbit polyclonal anti-**pS133-CREB** (**pCREB,** used at 1:250)	Millipore	Cat#06-519; RRID:AB_310153
Mouse monoclonal anti-**CtBP2** (IgG_1; used at 1:200)	BD Biosciences	Cat#612044; RRID:AB_399431
Mouse monoclonal anti-**GluR2** (IgG_{2a}, used at 1:1000)	Millipore	Cat#MAB397; RRID:AB_211385
Rabbit monoclonal anti-**GM130** (used at 1:500)	BD Biosciences	Cat# 610822; RRID:AB_398141
Rabbit polyclonal anti-**Myosin VIIA** (**MyoVIIa**, used at 1:150)	Proteus Biosciences	Cat#25-6790; RRID:AB_10015251
Mouse monoclonal anti-**Neurofilament 200 kDa** (**NF200**, used at 1:200)	Millipore	Cat#CBL212; RRID:AB_93408
Mouse monoclonal anti-**PSD-95** (IgG_{2a}; used at 1:500)	NeuroMab	Cat#75-028; RRID:AB_2877189
Rabbit polyclonal anti-**TrkB-FL (C-ter region)** (used at 1:500)	Abcam	Cat#ab18987; RRID:AB_444716
Goat anti-mouse IgG Alexa Fluor 546 (used at 1:500)	Invitrogen	Cat#A11003; RRID:AB_2534071
Goat anti-mouse IgG1 Alexa Fluor 488 (used at 1:500)	Invitrogen	Cat#A21121; RRID:AB_2535764
Goat anti-mouse IgG2a Alexa Fluor 555 (used at 1:500)	Invitrogen	Cat#A21137; RRID:AB_2535776
Goat anti-rabbit IgG Alexa Fluor 488 (used at 1:500)	Invitrogen	Cat#A11034; RRID:AB_2576217
Goat anti-rabbit IgG Pacific Blue (used at 1:200)	Invitrogen	Cat#P10994; RRID:AB_2539814
Cell-penetrating peptides		
**Bio-MTMyc** (Biotin-Ahx-*YGRKKRRQRRR*AEEQKLISEEDLLR-NH_2)	GenScript	N/A
**Bio-$MTFL_{457}$** (Biotin-Ahx-*YGRKKRRQRRR*HSKFGMKGPASVIS-NH_2)	GenScript	N/A
**MTMyc** (Ac-*YGRKKRRQRRR*AEEQKLISEEDLLR-NH_2)	GenScript	N/A
**$MTFL_{457}$** (Ac-*YGRKKRRQRRR*HSKFGMKGPASVIS-NH_2)	GenScript	N/A
Chemicals and culture products		
**Cytosine arabinoside** (**AraC**, used at 0.3 mg/ml)	Sigma-Aldrich	Cat#C1768; CAS: 147-94-4
**DAPI** (used at 1:1000)	Invitrogen	Cat#D1306
**Dulbecco's Modified Eagle's Medium/ Ham's Nutrient Mixture F12** (**DMEM-F12**)	Thermo Fisher Scientific	Cat#21331-020
**Ethylenediaminetetraacetic acid** (**EDTA,** used at 5%)	Sigma-Aldrich	Cat#E26290; CAS: 194491-31-1
**Fluorescein (FITC)-avidin** D (used at 1:30)	Vector Laboratories	Cat#A 2001
**Fluoro-Jade C** (used at 0.0004%)	Millipore	Cat#AG325
**Fluoromount-G**	SouthernBiotech	Cat#0100-01
D-**glucose** (used at 8.25 mM)	Sigma-Aldrich	Cat#G6152; CAS: 50-99-7
L-**glutamine** (used at 2 mM)	Thermo Fisher Scientific	Cat#25030-024
**Glycine** (used at 10 µM)	Bio-Rad	Cat#161-0718
**Goat serum**	Sigma-Aldrich	Cat#G9023

**Hank's Balanced Salt Solution** (**HBSS**)	Thermo Fisher Scientific	Cat#14025-050
**Insuline-Transferrine-Selenium 100X** (used at 1x)	Thermo Fisher Scientific	Cat#41400-045
**Kainate** (used at 500 µM)	Sigma-Aldrich	Cat#K0250;<br>CAS: 58002-62-3
**Ketamine** (**Ketamidor**, used at 100 mg/kg)	VetViva Richter GmbH	ATCvet:QN01AX03
**N2 supplement 100X** (used at 1x)	Thermo Fisher Scientific	Cat#41400-045
**NMDA** (used at 500 µM)	Tocris	Cat#0114;<br>CAS: 6384-92-5
**Phalloidin 647** (used at 1:200)	Invitrogen	Cat#A22287
**Paraformaldehyde** (**PFA**, used at 4%)	Sigma-Aldrich	Cat#158127<br>CAS: 30525-89-4
**Penicilin G** (used at 30 U/ml)	Sigma-Aldrich	Cat#P3032;<br>CAS: 69-57-8
**Pentobarbital sodium** (**Dolethal,** used at 150 mg/kg)	Vetoquinol	ATCvet:QN51AA01
**Sucrose**	Sigma-Aldrich	Cat#16104;<br>CAS: 57-50-1
**Tissue-Teck OCT**	Miles Diagnostic Division	Cat#4583
**TO-PRO3** (used at 1:1000)	Invitrogen	Cat#T3605
**Triton X-100**	Millipore	Cat#108603;<br>CAS: 9036-19-5
**Xylazine** (**Rompun,** used at 10 mg/kg)	Bayer	ATCvet:QN05CM92
Experimental Models: Organisms/Strains		
RjOrl:SWISS embryos mice (P2)	Janvier Labs, France	N/A
C57BL/6j mice	In site facility	N/A
Software		
BioSigRZ (https://www.tdt.com/)		RRID:SCR_014590
Fiji (http://fiji.sc)		RRID:SCR_002285
GraphPad Prism (http://www.graphpad.com/)		RRID:SCR_002798
Imaris (https://imaris.oxinst.com/)		RRID:SCR_007370
Other		
Disposable stainless steel needle electrodes	Technomed Medical	Cat#TE/S50716-001
6-well ThinCert™ cell culture inserts for multiwell plates	Greiner	Cat#657610
