# Supplementary material for: Neuroprotective potential of a TrkB-FL-derived cell-penetrating peptide in cochlear synaptopathy and noise-induced hearing loss
Source: Mol Med. 2026 Jun 29;32:148. doi: 10.1186/s10020-026-01539-9 (PMC13584400; doi:10.1186/s10020-026-01539-9)
Supplement: Supplementary file 2 [file 10020_2026_1539_MOESM2_ESM.pdf]

Supplementary Material 2

Neuroprotective potential of a TrkB-FL-derived cell-penetrating peptide in cochlear synaptopathy and noise-induced hearing loss

Authors:

Elena Torres-Campos[a], Isabel Varela-Nieto[a,b,c,*] and Margarita Díaz-Guerra[a,*]

Affiliations:

[a]Instituto de Investigaciones Biomédicas Sols-Morreale (IIBM), Consejo Superior de Investigaciones Científicas-Universidad Autónoma de Madrid, Madrid 28029, Spain

[b]Centro de Investigación Biomédica en Red en Enfermedades Raras (CIBERER), Instituto de Salud Carlos III (ISCIII), Madrid 28029, Spain

[c]Instituto de Investigación Sanitaria del Hospital La Paz (IdiPAZ), Madrid 28046, Spain.

*Corresponding authors: mdiazguerra@iib.uam.es (ORCID ID: 0000-0002-4478-0956) and i.varela.nieto@csic.es (ORCID ID: 0000-0003-3077-0500)

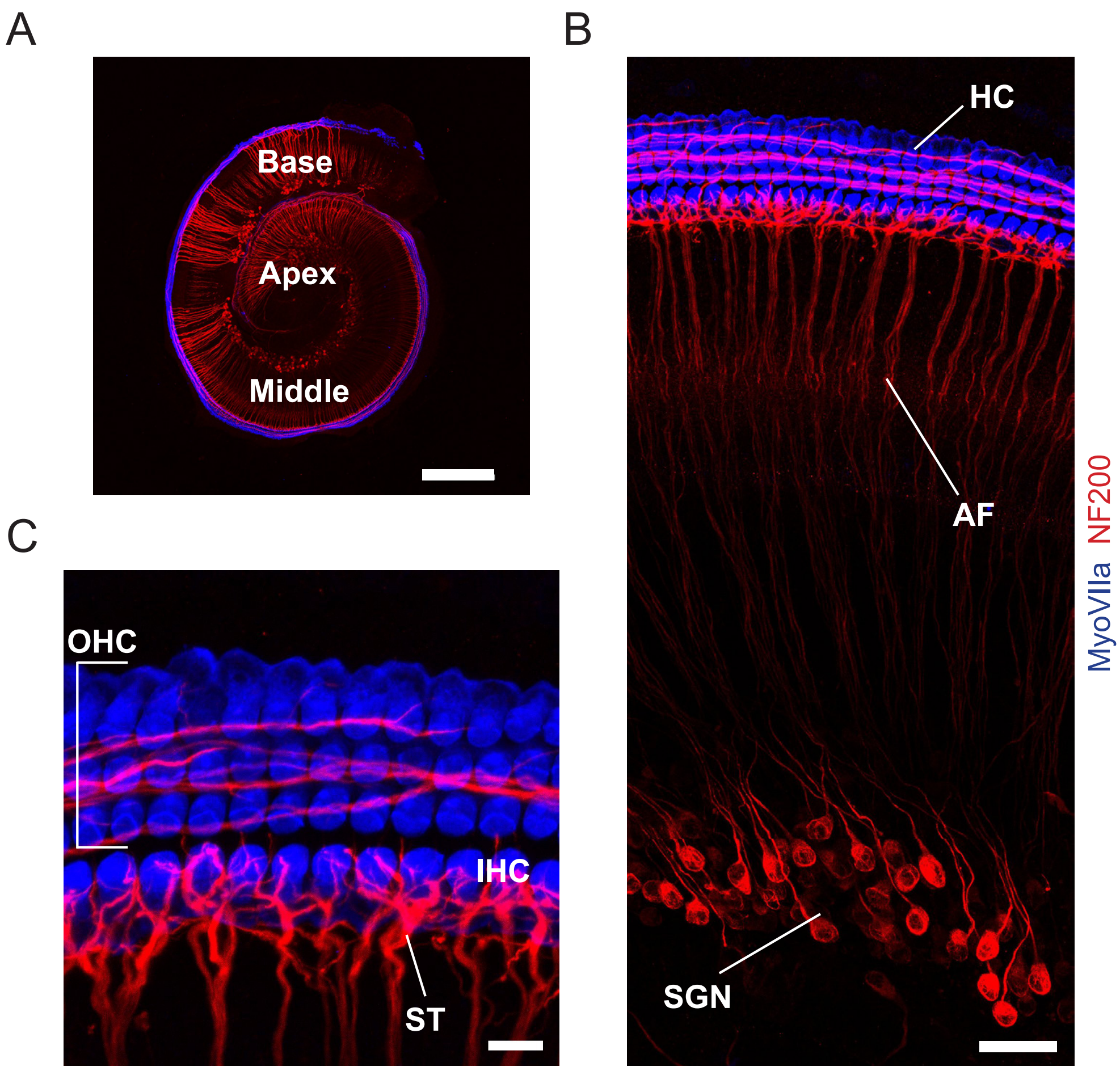

Supplementary Fig. 1 Ex vivo cochlear explants maintain the characteristic cellular architecture. **A** Representative confocal image of a whole cochlear explant showing the three cochlear regions: apex, middle, and base. Cochlear explants incubated for 48 h were fixed and labeled with antibodies for MyoVIIa (blue) and NF200 (red) to visualize, respectively, hair cells (HCs) and spiral ganglion neurons (SGNs). **B-C** Details of a cochlear explant at higher magnification illustrating the preservation of the characteristic cellular organization of the cochlea. **B** HCs innervated by afferent fibers (AF), originating from SGNs, can be clearly distinguished. **C** The spatial organization of the outer hair cells (OHCs) and inner hair cells (IHCs) is shown in detail, with OHCs organized in three rows and IHCs in a single row, the latter contacted by the synaptic terminals (ST) of SGNs. Scale bars: (**A**) 50 µm; (**B**) 20 µm; (**C**) 10 µm.

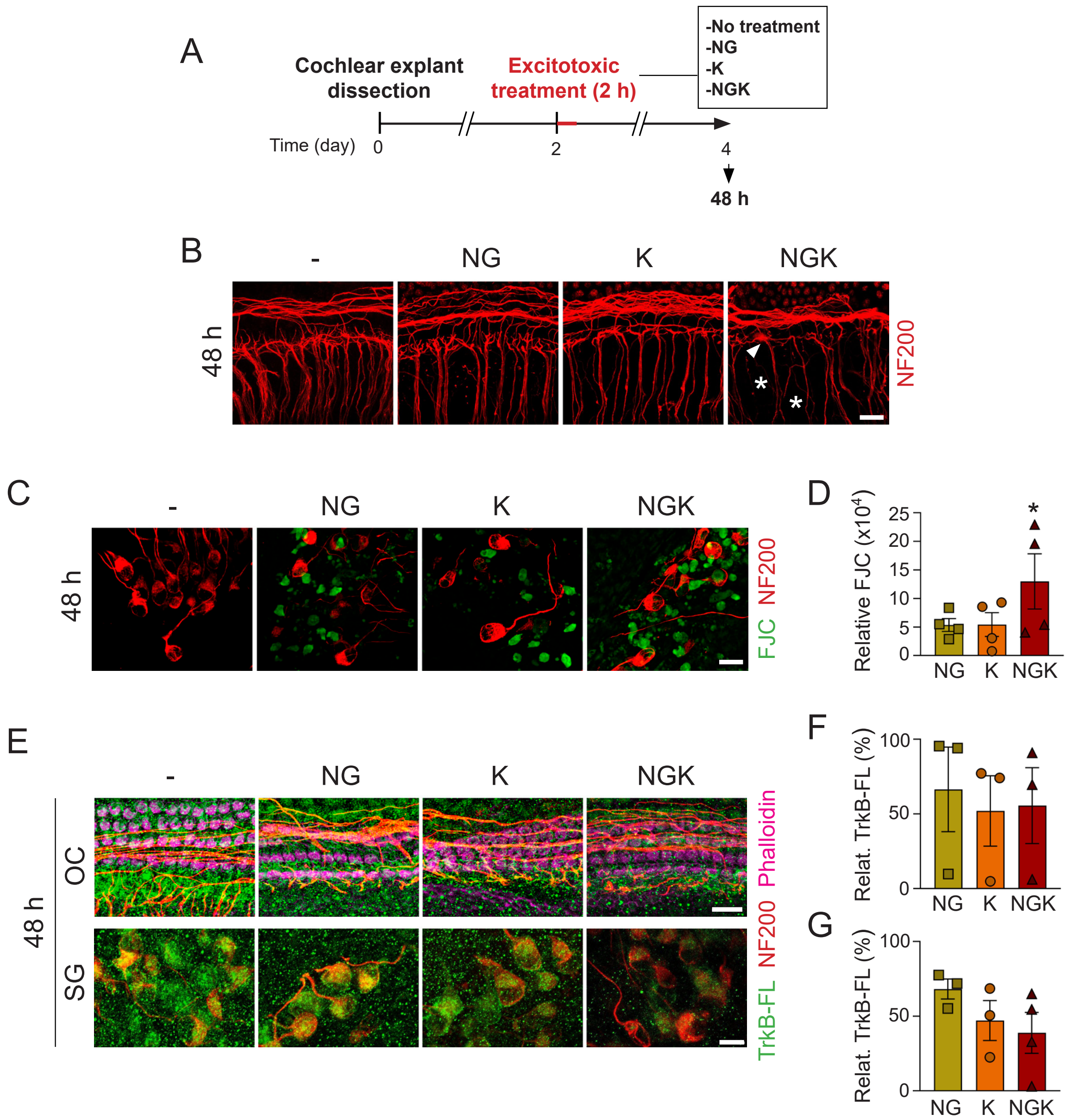

Supplementary Fig. 2 Excitotoxicity-induced neurodegeneration and TrkB-FL destabilization 48 h after damage induction. **A** Experimental design. Cochlear explants were treated for 2 h with different GluR agonists: NG, K or NGK. Subsequently, explants were fixed 48 h after excitotoxicity induction and processed for immunofluorescence, focusing on the cochlear middle turn. **B** Representative confocal images showing the excitotoxicity-induced degeneration of synaptic terminals and afferent fibers of SGNs, labeled for NF200 (red). Asterisk and arrowheads in NGK-treated explants indicate, respectively, the absence or presence of fibers and synaptic terminals. Qualitative analysis of the structural integrity of cochlear afferent innervation was performed using 3-4 explants per experimental condition. Scale bar: 20 µm. **C** Representative confocal images showing spiral ganglion neurodegeneration induced by different excitotoxic insults in explants stained with FJC (green) and labeled for NF200 (red). **D** Analysis of FJC marker levels, relative to the controls (untreated explants, assigned an arbitrary value of 1). Data are presented as mean ± SEM (n = 4 explants/condition). Statistically significant differences were established using Kruskal-Wallis test followed by Dunn's multiple comparison test: NGK vs control (* $P < 0.05$). **E** Representative confocal images showing the effect of different excitotoxic treatments on TrkB-FL receptor levels in the organ of Corti (OC, upper panel) and spiral ganglion (SG, lower panel). Explants were labeled for TrkB-FL (green), NF200 (red) and phalloidin (purple). Scale bars: 20 µm (upper panel); 10 µm (lower panel). **F-G** Evaluation of TrkB-FL levels, relative to the controls (untreated explants, assigned an arbitrary value of 100%), in the OC (**F**) and the SG (**G**) following the induction of the excitotoxic insults. Means ± SEM are represented (3-4 explants/condition). Statistical analysis was performed by Kruskal-Wallis test followed by Dunn's multiple comparison test (**F**) or one-way ANOVA test followed by Tukey's multiple comparison test (**G**). Although no significant differences were found, a tendency toward a decrease in TrkB-FL levels was observed in all the treatments.

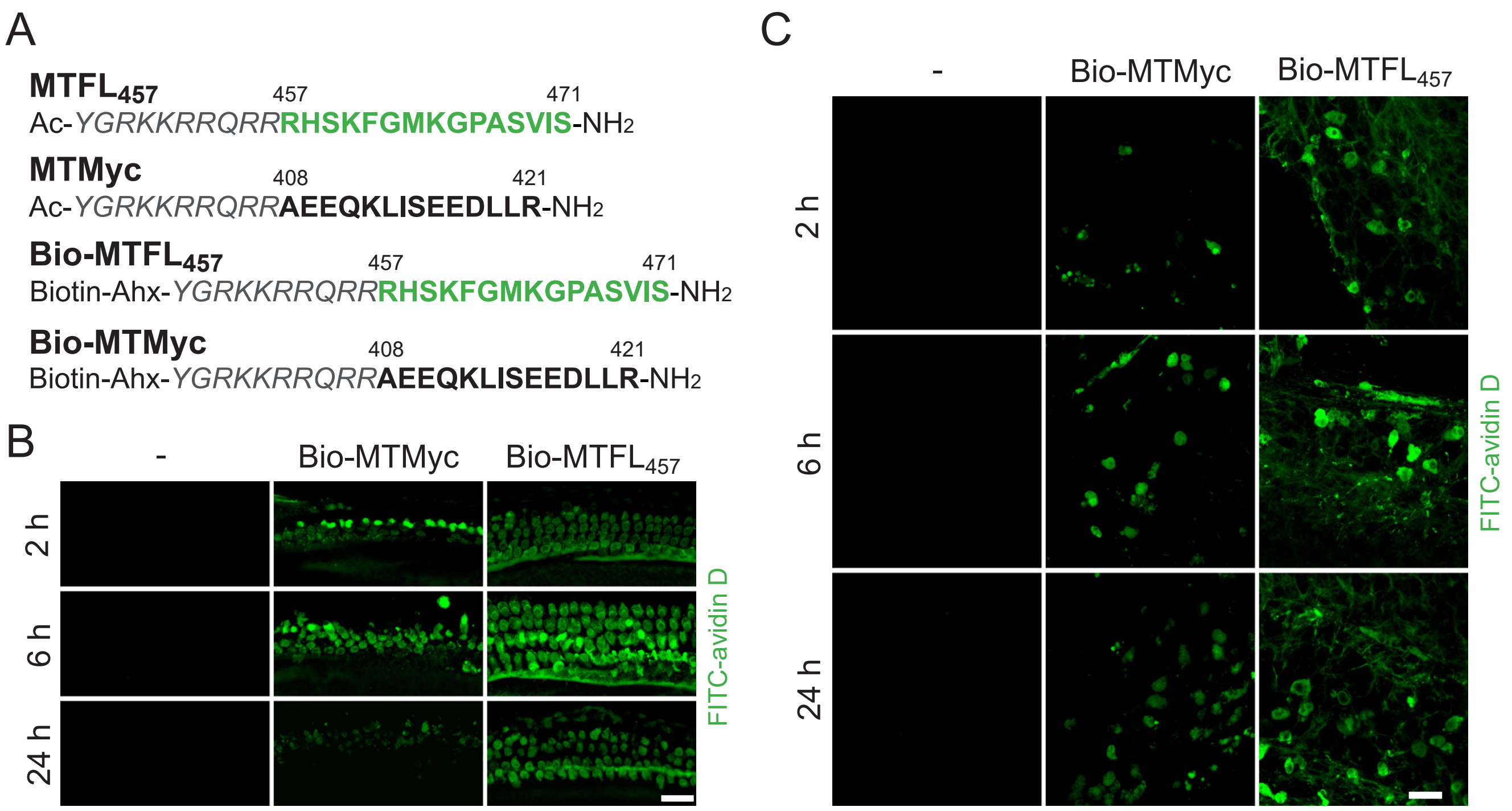

Supplementary Fig. 3 Designed CPPs penetrate and distribute through cochlear explants. **A** Sequence of peptides $MTFL_{457}$, MTMyc, Bio-$MTFL_{457}$, and Bio-MTMyc. The different peptides contain a short sequence belonging to protein Tat (aa 47–57; gray, italic) from HIV-1, followed by the indicated rat TrkB-FL (green, bold) or c-Myc (black, bold) sequences. The four CPPs contain an amido ($-NH_2$) at their C-ter. At the N-ter, $MTFL_{457}$ and MTMyc are modified by an acetyl (Ac-) group while their biotinylated forms, respectively Bio-$MTFL_{457}$ and Bio-MTMyc, contain a biotin moiety (Biotin–Ahx). **B-C** Representative confocal images of Bio-$MTFL_{457}$ and Bio-MTMyc biodistribution in the organ of Corti (**B**) and the spiral ganglion (**C**) within the middle cochlear turn. Explants were incubated with the biotinylated peptides (25 μM) for 2, 6 or 24 h and subsequently fixed and stained with fluorescein (FITC)-conjugated avidin D (green) for CPP detection. A qualitative analysis of Bio-$MTFL_{457}$ and Bio-MTMyc signal was performed from at least three explants per experimental condition. Scale bars: 20 μm.

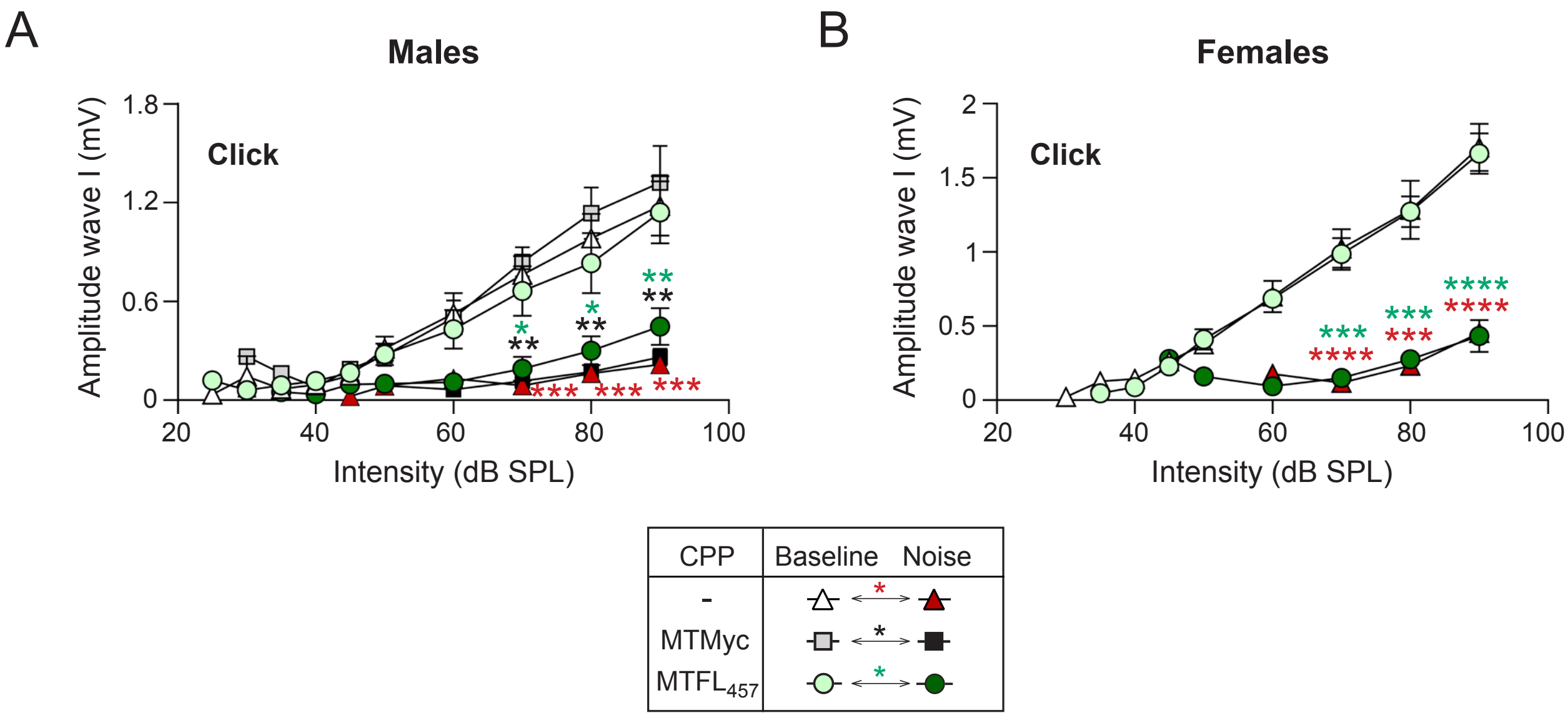

Supplementary Fig. 4 Effect of $MTFL_{457}$ on click-evoked wave I amplitude following noise exposure. Analysis of wave I amplitude plotted against click stimulus intensity (dB SPL) in male (**A**) and female (**B**) mice pre-treated or not with MTMyc (in the case of males) or $MTFL_{457}$ (10 nmol/g) before (baseline) and 1 day after noise exposure. Means ± SEM are represented (males, n = 6-11 animals/group; females, n = 4 animals/group). Statistical analysis for each intensity between the different groups was performed by one-way ANOVA test followed by Tukey's multiple comparison test: *, noise condition vs respective baseline in the absence of CPP (red), or in the presence of MTMyc (black) or $MTFL_{457}$ (green) (*, $P < 0.05$; **, $P < 0.01$; ***, $P < 0.001$; ****, $P < 0.0001$).
